# Thoracic and cardiovascular surgeries in Japan during 2023: Annual report by the Japanese Association for Thoracic Surgery

**DOI:** 10.1007/s11748-025-02128-z

**Published:** 2025-05-16

**Authors:** Committee for Scientific Affairs, The Japanese Association for Thoracic Surgery, Naoki Yoshimura, Yukio Sato, Hiroya Takeuchi, Tomonobu Abe, Toshio Doi, Toyofumi Fengshi Yoshikawa, Yasutaka Hirata, Michiko Ishida, Hisashi Iwata, Takashi Kamei, Nobuyoshi Kawaharada, Shunsuke Kawamoto, Kohji Kohno, Kazuo Koyanagi, Hiraku Kumamaru, Goro Matsumiya, Kenji Minatoya, Noboru Motomura, Rie Nakahara, Morihito Okada, Hisashi Saji, Aya Saito, Kenji Suzuki, Hirofumi Takemura, Yasue Kimura, Wataru Tatsuishi, Hiroyuki Yamamoto, Takushi Yasuda, Hideyuki Shimizu, Masayuki Chida

**Affiliations:** 1 The Japanese Association for Thoracic Surgery, Committee for Scientific Affairs, Tokyo, Japan; 2 Department of Thoracic and Cardiovascular Surgery, Graduate School of Medicine, University of Toyama, Toyama, Japan; 3 Department of Thoracic Surgery, University of Tsukuba, Tsukuba, Japan; 4 Department of Surgery, Hamamatsu University School of Medicine, Shizuoka, Japan; 5 Division of Cardiovascular Surgery, Department of General Surgical Science, Gunma University, Maebashi, Japan; 6 Department of Thoracic Surgery, Nagoya University Graduate School of Medicine, Nagoya, Japan; 7 Department of Cardiovascular Surgery, National Center for Child Health and Development, Tokyo, Japan; 8 Cardiac Surgery, Handa City Hospital, Aichi, Japan; 9 Department of General Thoracic Surgery, Gifu University Hospital, Gifu, Japan; 10 Department of Surgery, Graduate School of Medicine, Tohoku University, Sendai, Japan; 11 Department of Cardiovascular Surgery, Sapporo Medical University School of Medicine, Sapporo, Japan; 12 Department of Cardiovascular Surgery, Tohoku Medical and Pharmaceutical University Hospital, Sendai, Japan; 13 Department of Gastrointestinal Tract Surgery, Fukushima Medical University, Fukushima, Japan; 14 Department of Gastroenterological Surgery, Tokai University School of Medicine, Isehara, Japan; 15 Department of Healthcare Quality Assessment, Graduate School of Medicine, The University of Tokyo, Tokyo, Japan; 16 Department of Cardiovascular Surgery, Chiba University Graduate School of Medicine, Chiba, Japan; 17 Department of Cardiovascular Surgery, Graduate School of Medicine, Kyoto University, Kyoto, Japan; 18 Department of Cardiovascular Surgery, Toho University Sakura Medical Center, Chiba, Japan; 19 Division of Thoracic Surgery, Tochigi Cancer Center, Tochigi, Japan; 20 Surgical Oncology, Hiroshima University, Hiroshima, Japan; 21 Department of Chest Surgery, St. Marianna University School of Medicine, Kawasaki, Japan; 22 Department of Surgery, Graduate School of Medicine, Yokohama City University, Yokohama, Japan; 23 Department of General Thoracic Surgery, Juntendo University School of Medicine, Tokyo, Japan; 24 Department of Cardiovascular Surgery, Kanazawa University, Kanazawa, Japan; 25 Department of Gastroenterological Surgery, National Hospital Organization Kyushu Cancer Center, Fukuoka, Japan; 26 Department of Surgery, Faculty of Medicine, Kindai University, Osaka, Japan; 27 Department of Cardiovascular Surgery, Keio University, Tokyo, Japan; 28 Department of General Thoracic Surgery, Dokkyo Medical University, Tochigi, Japan

Since 1986, the Japanese Association for Thoracic Surgery (JATS) has conducted annual thoracic surgery surveys throughout Japan to determine statistics on the number of procedures performed by surgical categories. Herein, we summarize the results of the association’s annual thoracic surgery surveys in 2023.

Adhering to the norm thus far, thoracic surgery has been classified into three categories, including cardiovascular, general thoracic, and esophageal surgeries, with patient data for each group being examined and analyzed. We honor and value all members’ continued professional support and contributions.

Incidence of hospital mortality was included in the survey to determine nationwide status, which has contributed to Japanese surgeons’ understanding of the present status of thoracic surgery in Japan while helping in surgical outcome improvements by enabling comparisons between their work and that of others. This approach has enabled the association to gain a better understanding of present problems and prospects, which is reflected in its activities and member education.

The 30-day mortality (also known as *operative mortality*) is defined as death within 30 days of surgery, regardless of the patient’s geographic location, including post-discharge from the hospital. *Hospital mortality* is defined as death within any time interval following surgery among patients yet to be discharged from the hospital.

Transfer to a nursing home or a rehabilitation unit is considered hospital discharge unless the patient subsequently dies of complications from surgery, while hospital-to-hospital transfer during esophageal surgery is not considered a form of discharge. In contrast, hospital-to-hospital transfer 30 days following cardiovascular and general thoracic surgeries are considered discharge given that National Clinical Database (NCD)-related data were used in these categories.

Severe Acute Respiratory Syndrome Coronavirus-2 (SARS-CoV-2), the causative pathogen for the coronavirus disease 2019 (COVID-19), first emerged in Wuhan, China, in December 2019, and by March 2020, it was declared a pandemic [1]. The pandemic of SARS-CoV-2 resulted in a global healthcare and financial crisis. There was a significant estimated reduction in the national case volume of cardiovascular, general thoracic, and esophageal surgeries in Japan from 2020 to 2022 [2–6]. We have to continue the estimation of the nationwide effect of SARS-CoV-2 pandemic on thoracic surgery in Japan, with surgical volume, outcomes and patient data for each group.

## Survey abstract

All data on cardiovascular, general thoracic, and esophageal surgeries were obtained from the NCD. In 2018, the data collection method for general thoracic and esophageal surgeries had been modified from self-reports using questionnaire sheets following each institution belonging to the JATS to an automatic package downloaded from the NCD in Japan.

The data collection related to cardiovascular surgery (initially self-reported using questionnaire sheets in each participating institution up to 2014) changed to downloading an automatic package from the Japanese Cardiovascular Surgery Database (JCVSD), which is a cardiovascular subsection of the NCD in 2015.

## Final report: 2023

### (A) Cardiovascular surgery

We are extremely pleased with the cooperation of our colleagues (members) in completing the cardiovascular surgery survey, which has undoubtedly improved the quality of this annual report. We are truly grateful for the significant efforts made by all participants within each participating institution in completing the JCVSD/NCD.

**Fig. 1:**
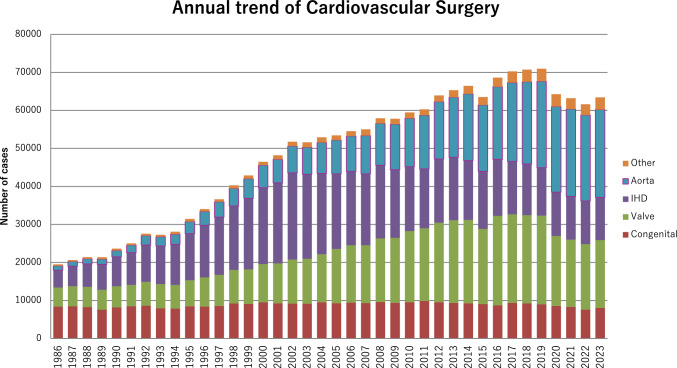
Annual trend of cardiovascular surgery

Figure 1 illustrates the development of cardiovascular surgery in Japan over the past 35 years. Aortic surgery includes only surgeries for aortic dissection and thoracic and thoracoabdominal aortic aneurysms. Extra-anatomic bypass surgery for thoracic aneurysm and pacemaker implantation have been excluded from the survey since 2015. Ventricular assist device (VAD) implantations had not been included in the total number of surgical procedures but we have decided to count the number of VAD implantations from this time. VAD implantations since 2016 are added to Fig. 1.

A total of 63,427 cardiovascular surgeries, including 150 VAD implantations and 115 heart transplants, had been performed in 2023, with a 3.0% increase compared to that in 2022 (*n* = 61,606). Following on from 2020, a decline in the number of cases has been observed for the third consecutive year. In 2023, the downward trend finally came to a halt and started to increase. As the issues related to COVID-19 are being resolved, a gradual recovery in the number of surgeries is expected in the future.

Compared to data for 2022 [5] and 2013 [7], data for 2023 showed 6.6% (8084 vs. 7580) more and 14.7% fewer surgeries for congenital heart disease, 3.2% (17,805 vs. 17,260) more and 18.2% fewer surgeries for valvular heart disease, 1.0% (11,227 vs. 11,340) and 32.3% fewer surgeries for ischemic heart procedures, and 2.2% (23,104 vs. 22,597) and 46.6% more surgeries for thoracic aorta, respectively. Data for individual categories are summarized in Tables 1, 2, 3, 4, 5 and 6.

**Table 1:** Congenital (total; 8084) (1)CPB ( +) (total; 6190)

	Neonate				Infant				1–17 years				≥ 18 years				Total			
	Cases	30-Day mortality		Hospital mortality	Cases	30-Day mortality		Hospital mortality	Cases	30-Day mortality		Hospital mortality	Cases	30-Day mortality		Hospital mortality	Cases	30-Day mortality		Hospital mortality
		Hospital	After discharge			Hospital	After discharge			Hospital	After discharge			Hospital	After discharge			Hospital	After discharge	
PDA	5	2 (40.0)	0	2 (40.0)	2	0	0	0	1	0	0	0	12	0	0	0	20	2 (10.0)	0	2 (10.0)
Coarctation (simple)	4	0	0	0	6	0	0	0	14	0	0	0	3	0	0	0	27	0	0	0
+ VSD	41	0	0	0	46	0	0	1 (2.2)	26	0	0	1 (3.8)	2	0	0	0	115	0	0	2 (1.7)
+ DORV	3	0	0	1 (33.3)	3	0	0	0	2	0	0	0	0	0	0	0	8	0	0	1 (12.5)
+ AVSD	6	0	0	1 (16.7)	6	0	0	0	3	0	0	0	0	0	0	0	15	0	0	1 (6.7)
+ TGA	0	0	0	0	0	0	0	0	0	0	0	0	0	0	0	0	0	0	0	0
+ SV	2	0	0	0	4	0	0	0	2	0	0	0	0	0	0	0	8	0	0	0
+ Others	4	0	0	0	8	0	0	0	3	0	0	0	1	0	0	0	16	0	0	0
Interrupt. of Ao (simple)	0	0	0	0	0	0	0	0	0	0	0	0	0	0	0	0	0	0	0	0
+ VSD	12	0	0	0	25	1 (4.0)	0	2 (8.0)	6	0	0	0	1	0	0	0	44	1 (2.3)	0	2 (4.5)
+ DORV	0	0	0	0	1	0	0	1 (100.0)	1	0	0	0	0	0	0	0	2	0	0	1 (50.0)
+ Truncus	4	0	0	0	3	0	0	0	1	0	0	0	0	0	0	0	8	0	0	0
+ TGA	0	0	0	0	0	0	0	0	0	0	0	0	0	0	0	0	0	0	0	0
+ Others	1	0	0	0	2	0	0	0	3	0	0	0	0	0	0	0	6	0	0	0
Vascular ring	0	0	0	0	1	0	0	0	2	0	0	0	0	0	0	0	3	0	0	0
PS	2	0	0	0	16	0	0	0	67	0	0	0	17	0	0	0	102	0	0	0
PA⋅IVS or Critical PS	8	0	0	0	33	0	0	0	44	0	0	0	7	0	0	0	92	0	0	0
TAPVR	85	3 (3.5)	0	7 (8.2)	49	0	0	1 (2.0)	22	0	0	0	2	0	0	0	158	3 (1.9)	0	8 (5.1)
PAPVR ± ASD	0	0	0	0	2	0	0	1 (50.0)	43	0	0	0	13	0	0	0	58	0	0	1 (1.7)
ASD	2	0	0	0	31	0	0	0	436	0	0	0	821	11 (1.3)	0	0	1290	11 (0.9)	0	11 (0.9)
Cor triatriatum	0	0	0	0	4	0	0	0	2	0	0	0	1	0	0	0	7	0	0	0
AVSD (partial)	0	0	0	0	13	1 (7.7)	0	1 (7.7)	37	0	0	0	6	0	0	0	56	1 (1.8)	0	1 (1.8)
AVSD (complete)	4	0	0	0	77	0	0	0	104	0	0	0	4	1 (25.0)	0	1 (25.0)	189	1 (0.5)	0	1 (0.5)
+ TOF or DORV	0	0	0	0	4	0	0	0	8	0	0	0	1	0	0	0	13	0	0	0
+ Others	0	0	0	0	0	0	0	0	0	0	0	0	0	0	0	0	0	0	0	0
VSD (subarterial)	2	0	0	0	84	0	0	0	133	0	0	0	9	0	0	0	228	0	0	0
VSD (perimemb./muscular)	8	0	0	0	563	0	0	0	340	0	0	0	21	0	0	0	932	0	0	0
VSD (type unknown)	0	0	0	0	0	0	0	0	6	0	0	0	146	3 (2.1)	0	3 (2.1)	152	3 (2.0)	0	3 (2.0)
VSD + PS	0	0	0	0	18	0	0	0	13	0	0	0	0	0	0	0	31	0	0	0
DCRV ± VSD	1	0	0	0	4	0	0	0	11	0	0	0	14	0	0	0	30	0	0	0
Aneurysm of sinus of valsalva	0	0	0	0	0	0	0	0	1	0	0	0	4	0	0	0	5	0	0	0
TOF	8	0	0	0	145	0	0	1 (0.7)	155	0	0	0	49	1 (2.0)	0	1 (2.0)	357	1 (0.3)	0	2 (0.6)
PA + VSD	3	0	0	0	69	0	1 (1.4)	0	86	1 (1.2)	0	2 (2.3)	9	0	0	0	167	1 (0.6)	1 (0.6)	2 (1.2)
DORV	14	0	0	0	134	2 (1.5)	0	3 (2.2)	109	0	0	1 (0.9)	8	0	0	0	265	2 (0.8)	0	4 (1.5)
TGA (simple)	62	2 (3.2)	0	4 (6.5)	8	0	0	0	7	0	0	0	1	0	0	0	78	2 (2.6)	0	4 (5.1)
+ VSD	21	0	0	1 (4.8)	10	1 (10.0)	0	1 (10.0)	16	0	0	0	0	0	0	0	47	1 (2.1)	0	2 (4.3)
VSD + PS	0	0	0	0	0	0	0	0	0	0	0	0	0	0	0	0	0	0	0	0
Corrected TGA	5	0	0	1 (20.0)	16	0	0	0	36	0	0	0	7	0	0	0	64	0	0	1 (1.6)
Truncus arteriosus	5	0	0	0	16	0	0	0	20	0	0	0	3	0	0	0	44	0	0	0
SV	20	5 (25.0)	0	6 (30.0)	174	3 (1.7)	0	7 (4.0)	135	2 (1.5)	0	2 (1.5)	24	1 (4.2)	0	1 (4.2)	353	11 (3.1)	0	16 (4.5)
TA	4	0	0	0	40	0	0	2 (5.0)	44	0	0	0	5	1 (20.0)	0	1 (20.0)	93	1 (1.1)	0	3 (3.2)
HLHS	16	3 (18.8)	0	4 (25.0)	100	2 (2.0)	0	7 (7.0)	61	1 (1.6)	0	2 (3.3)	2	0	0	0	179	6 (3.4)	0	13 (7.3)
Aortic valve lesion	6	0	0	0	14	1 (7.1)	0	1 (7.1)	67	0	0	0	38	0	0	0	125	1 (0.8)	0	1 (0.8)
Mitral valve lesion	1	1 (100.0)	0	1 (100.0)	21	0	0	0	58	0	0	0	22	0	0	0	102	1 (1.0)	0	1 (1.0)
Ebstein	8	1 (12.5)	0	2 (25.0)	11	1 (9.1)	0	1 (9.1)	29	0	0	1 (3.4)	15	0	0	0	63	2 (3.2)	0	4 (6.3)
Coronary disease	0	0	0	0	17	0	0	0	12	0	0	0	5	0	0	0	34	0	0	0
Others	5	0	0	2 (40.0)	17	0	0	1 (5.9)	35	1 (2.9)	0	1 (2.9)	232	3 (1.3)	0	3 (1.3)	289	4 (1.4)	0	7 (2.4)
Conduit failure	0	0	0	0	0	0	0	0	24	0	0	0	11	0	0	1 (9.1)	35	0	0	1 (2.9)
Redo (excluding conduit failure)	2	0	0	0	60	5 (8.3)	0	8 (13.3)	106	2 (1.9)	0	3 (2.8)	112	1 (0.9)	0	2 (1.8)	280	8 (2.9)	0	13 (4.6)
Total	374	17 (4.5)	0	32 (8.6)	1857	17 (0.9)	1 (0.05)	39 (2.1)	2331	7 (0.3)	0	13 (0.6)	1628	22 (1.4)	0	13 (0.8)	6190	63 (1.0)	1 (0.0)	108 (1.7)
(), %mortality																				
*CPB* cardiopulmonary bypass; *PDA* patent ductus arteriosus; *VSD* ventricular septal defect; *DORV* double outlet right ventricle; *AVSD* atrioventricular septal defect; *TGA* transposition of great arteries; *SV* single ventricle; *Interrupt. of Ao.* interruption of aorta; *PS* pulmonary stenosis; *PA-IVS* pulmonary atresia within tact ventricular septum; *TAPVR* total anomalous pulmonary venous return; *PAPVR* partial anomalous pulmonary venous return; *ASD* atrial septal defect; *TOF* tetralogy of Fallot; *DCRV* double-chambered right ventricle; *TA* tricuspid atresia; *HLHS* hypoplastic left heart syndrome; *RV-PA* right ventricle-pulmonary artery																				

(2) CPB (-) (total; 1894)																				
	Neonate				Infant				1–17 years				≥ 18 years				Total			
	Cases	30-Day mortality		Hospital mortality	Cases	30-Day mortality		Hospital mortality	Cases	30-Day mortality		Hospital mortality	Cases	30-Day mortality		Hospital mortality	Cases	30-Day mortality		Hospital mortality
		Hospital	After discharge			Hospital	After discharge			Hospital	After discharge			Hospital	After discharge			Hospital	After discharge	
PDA	230	3 (1.3)	0	7 (3.0)	91	0	0	1 (1.1)	5	0	0	0	1	0	0	0	327	3 (0.9)	0	8 (2.4)
Coarctation (simple)	10	0	0	0	14	0	0	0	3	0	0	0	0	0	0	0	27	0	0	0
+ VSD	47	0	0	0	13	1 (7.7)	0	1 (7.7)	0	0	0	0	0	0	0	0	60	1 (1.7)	0	1 (1.7)
+ DORV	6	0	0	2 (33.3)	1	0	0	0	0	0	0	0	0	0	0	0	7	0	0	2 (28.6)
+ AVSD	4	0	0	0	0	0	0	0	0	0	0	0	0	0	0	0	4	0	0	0
+ TGA	0	0	0	0	0	0	0	0	0	0	0	0	0	0	0	0	0	0	0	0
+ SV	1	0	0	0	0	0	0	0	0	0	0	0	0	0	0	0	1	0	0	0
+ Others	4	0	0	0	6	0	0	1 (16.7)	1	0	0	0	0	0	0	0	11	0	0	1 (9.1)
Interrupt. of Ao (simple)	0	0	0	0	0	0	0	0	0	0	0	0	0	0	0	0	0	0	0	0
+ VSD	21	1 (4.8)	0	2 (9.5)	5	0	0	0	2	0	0	1 (50.0)	0	0	0	0	28	1 (3.6)	0	3 (10.7)
+ DORV	0	0	0	0	0	0	0	0	0	0	0	0	0	0	0	0	0	0	0	0
+ Truncus	8	0	0	0	1	0	0	0	0	0	0	0	0	0	0	0	9	0	0	0
+ TGA	0	0	0	0	0	0	0	0	0	0	0	0	0	0	0	0	0	0	0	0
+ Others	3	0	0	0	1	0	0	0	1	0	0	0	0	0	0	0	5	0	0	0
Vascular ring	8	0	0	0	20	0	0	0	17	0	0	0	0	0	0	0	45	0	0	0
PS	3	0	0	0	5	0	0	1 (20.0)	1	0	0	0	1	0	0	0	10	0	0	1 (10.0)
PA⋅IVS or Critical PS	10	0	0	0	14	0	0	0	4	2 (50.0)	0	2 (50.0)	0	0	0	0	28	2 (7.1)	0	2 (7.1)
TAPVR	11	1 (9.1)	0	2 (18.2)	6	0	0	1 (16.7)	1	0	0	0	0	0	0	0	18	1 (5.6)	0	3 (16.7)
PAPVR ± ASD	0	0	0	0	6	0	0	1 (16.7)	0	0	0	0	1	0	0	0	7	0	0	1 (14.3)
ASD	0	0	0	0	1	0	0	0	4	0	0	0	8	0	0	0	13	0	0	0
Cor triatriatum	0	0	0	0	0	0	0	0	0	0	0	0	0	0	0	0	0	0	0	0
AVSD (partial)	3	1 (33.3)	0	1 (33.3)	0	0	0	0	0	0	0	0	3	0	0	0	6	1 (16.7)	0	1 (16.7)
AVSD (complete)	29	0	0	0	52	1 (1.9)	0	2 (3.8)	11	0	0	0	7	1 (14.3)	0	1 (14.3)	99	2 (2.0)	0	3 (3.0)
+ TOF or DORV	3	0	0	0	10	0	0	0	3	0	0	0	0	0	0	0	16	0	0	0
+ Others	0	0	0	0	0	0	0	0	0	0	0	0	0	0	0	0	0	0	0	0
VSD (subarterial)	1	0	0	0	5	0	0	0	0	0	0	0	0	0	0	0	6	0	0	0
VSD (perimemb./muscular)	61	0	0	0	135	3 (2.2)	0	5 (3.7)	5	0	0	0	0	0	0	0	201	3 (1.5)	0	5 (2.5)
VSD (Type Unknown)	0	0	0	0	1		0	0	0	0	0	0	1	0	0	0	2	0	0	0
VSD + PS	0	0	0	0	1	0	0	0	0	0	0	0	0	0	0	0	1	0	0	0
DCRV ± VSD	0	0	0	0	0	0	0	0	0	0	0	0	0	0	0	0	0	0	0	0
Aneurysm of sinus of Valsalva	0	0	0	0	0	0	0	0	0	0	0	0	0	0	0	0	0	0	0	0
TOF	10	0	0	0	53	0	0	0	8	0	0	0	8	0	0	0	79	0	0	0
PA + VSD	7	0	0	1 (14.3)	31	0	0	0	18	0	0	0	3	0	0	0	59	0	0	1 (1.7)
DORV	39	0	0	1 (2.6)	59	2 (3.4)	0	2 (3.4)	11	0	0	0	0	0	0	0	109	2 (1.8)	0	3 (2.8)
TGA (simple)	7	0	0	0	5	1 (20.0)	0	2 (40.0)	3	0	0	0	1	0	0	0	16	1 (6.3)	0	2 (12.5)
+ VSD	11	0	0	3 (27.3)	1	0	0	0	2	0	0	0	0	0	0	0	14	0	0	3 (21.4)
VSD + PS	0	0	0	0	0	0	0	0	0	0	0	0	0	0	0	0	0	0	0	0
Corrected TGA	4	0	0	0	18	0	0	1 (5.6)	7	0	0	0	1	0	0	0	30	0	0	1 (3.3)
Truncus arteriosus	20	0	0	1 (5.0)	5	0	0	0	4	0	0	0	0	0	0	0	29	0	0	1 (3.4)
SV	44	3 (6.8)	0	6 (13.6)	65	3 (4.6)	0	4 (6.2)	25	1 (4.0)	0	1 (4.0)	10	0	0	1 (10.0)	144	7 (4.9)	0	12 (8.3)
TA	15	0	0	0	13	1 (7.7)	0	2 (15.4)	4	0	0	0	4	0	0	0	36	1 (2.8)	0	2 (5.6)
HLHS	76	1 (1.3)	0	7 (9.2)	32	1 (3.1)	0	1 (3.1)	17	1 (5.9)	0	2 (11.8)	1	0	0	0	126	3 (2.4)	0	10 (7.9)
Aortic valve lesion	3	0	0	0	1	0	0	0	10	1 (10.0)	0	1 (10.0)	0	0	0	0	14	1 (7.1)	0	1 (7.1)
Mitral valve lesion	0	0	0	0	1	0	0	0	3	0	0	0	0	0	0	0	4	0	0	0
Ebstein	4	1 (25.0)	0	1 (25.0)	1	1 (100.0)	0	1 (100.0)	6	1 (16.7)	0	1 (16.7)	1	0	0	0	12	3 (25.0)	0	3 (25.0)
Coronary disease	0	0	0	0	0	0	0	0	1	0	0	0	0	0	0	0	1	0	0	0
Others	8	1 (12.5)	0	1 (12.5)	10	2 (20.0)	0	4 (40.0)	20	7 (35.0)	0	8 (40.0)	9	0	0	0	47	10 (21.3)	0	13 (27.7)
Conduit failure	0	0	0	0	0	0	0	0	0	0	0	0	0	0	0	0	0	0	0	0
Redo (excluding conduit failure)	14	2 (14.3)	0	2 (14.3)	91	1 (1.1)	0	6 (6.6)	115	3 (2.6)	0	5 (4.3)	23	1 (4.3)	0	3 (13.0)	243	7 (2.9)	0	16 (6.6)
Total	725	14 (1.9)	0	37 (5.1)	774	17 (2.2)	0	36 (4.7)	312	16 (5.1)	0	21 (6.7)	83	2 (2.4)	0	5 (6.0)	1894	49 (2.6)	0	99 (5.2)
(), % mortality																				
*CPB* cardiopulmonary bypass; *PDA* patent ductus arteriosus; *VSD* ventricular septal defect; *DORV* double outlet right ventricle; *AVSD* atrioventricular septal defect; *TGA* transposition of the great arteries; *SV* single ventricle; *Interrupt. of Ao*., interruption of aorta; *PS* pulmonary stenosis; *PA-IVS* pulmonary atresia with intact ventricular septum; *TAPVR* total anomalous pulmonary venous return; *PAPVR* partial anomalous pulmonary venous return; *ASD* atrial septal defect; *TOF* tetralogy of Fallot; *DCRV* double-chambered right ventricle; *TA* tricuspid atresia; *HLHS* hypoplastic left heart syndrome; *RV-PA* right ventricle-pulmonary artery																				

(3) Main procedure																					
		Neonate				Infant				1- 17 years				≥ 18 years				Total			
		Cases	30-Day mortality		Hospital mortality	Cases	30-Day mortality		Hospital mortality	Cases	30-Day mortality		Hospital mortality	Cases	30-Day mortality		Hospital mortality	Cases	30-Day mortality		Hospital mortality
				After discharge			Hospital	After discharge			Hospital	After discharge			Hospital	After discharge			Hospital	After discharge	
1	SP Shunt	81	1 (1.2)	0	6 (7.4)	317	2 (0.6)	1 (0.3)	5 (1.6)	41	0	0	1 (2.4)	0	0	0	0	439	3 (0.7)	1 (0.2)	12 (2.7)
2	PAB	233	3 (1.3)	0	12 (5.2)	260	3 (1.2)	0	7 (2.7)	12	0	0	0	2	0	0	0	507	6 (1.2)	0	19 (3.7)
3	Bidirectional Glenn or hemi-Fontan ± α	0	0	0	0	213	2 (0.9)	0	3 (1.4)	70	0	0	0	2	0	0	0	285	2 (0.7)	0	3 (1.1)
4	Damus–Kaye–Stansel operation	0	0	0	0	14	1 (7.1)	0	2 (14.3)	11	0	0	0	1	0	0	0	26	1 (3.8)	0	2 (7.7)
5	PA reconstruction/repair (including redo)	15	0	0	0	154	0	0	3 (1.9)	195	0	0	2 (1.0)	28	0	0	0	392	0	0	5 (1.3)
6	RVOT reconstruction/repair	6	1 (16.7)	0	1 (16.7)	180	0	0	2 (1.1)	282	1 (0.4)	0	1 (0.4)	33	0	0	0	501	2 (0.4)	0	4 (0.8)
7	Rastelli procedure	1	0	0	0	45	2 (4.4)	0	2 (4.4)	73	1 (1.4)	0	1 (1.4)	4	0	0	0	123	3 (2.4)	0	3 (2.4)
8	Arterial switch procedure	92	3 (3.3)	0	7 (7.6)	20	1 (5.0)	0	1 (5.0)	5	0	0	1 (20.0)	0	0	0	0	117	4 (3.4)	0	9 (7.7)
9	Atrial switch procedure	0	0	0	0	1	0	0	0	2	0	0	0	2	0	0	0	5	0	0	0
10	Double switch procedure	0	0	0	0	1	0	0	0	4	0	0	0	0	0	0	0	5	0	0	0
11	Repair of anomalous origin of CA	0	0	0	0	7	0	0	0	3	0	0	0	1	0	0	0	11	0	0	0
12	Closure of coronary AV fistula	1	0	0	0	4	0	0	0	0	0	0	0	4	0	0	0	9	0	0	0
13	Fontan / TCPC	0	0	0	0	0	0	0	0	273	4 (1.5)	0	4 (1.5)	25	1 (4.0)	0	1 (4.0)	298	5 (1.7)	0	5 (1.7)
14	Norwood procedure	17	1 (5.9)	0	2 (11.8)	98	4 (4.1)	0	9 (9.2)	2	0	0	0	0	0	0	0	117	5 (4.3)	0	11 (9.4)
15	Ventricular septation	0	0	0	0	0	0	0	0	2	0	0	0	0	0	0	0	2	0	0	0
16	Left side AV valve repair (including Redo)	2	1 (50.0)	0	1 (50.0)	25	0	0	0	66	0	0	0	22	0	0	0	115	1 (0.9)	0	1 (0.9)
17	Left side AV valve replace (including Redo)	0	0	0	0	7	0	0	0	31	0	0	0	19	2 (10.5)	0	2 (10.5)	57	2 (3.5)	0	2 (3.5)
18	Right side AV valve repair (including Redo)	12	1 (8.3)	0	2 (16.7)	110	2 (1.8)	0	3 (2.7)	94	1 (1.1)	0	2 (2.1)	75	0	0	0	291	4 (1.4)	0	7 (2.4)
19	Right side AV valve replace (including Redo)	1	0	0	0	1	0	0	0	8	0	0	1 (12.5)	40	0	0	0	50	0	0	1 (2.0)
20	Common AV valve repair (including Redo)	2	2 (100.0)	0	2 (100.0)	23	1 (4.3)	0	5 (21.7)	12	0	0	0	2	0	0	0	39	3 (7.7)	0	7 (17.9)
21	Common AV valve replace (including Redo)	1	0	0	0	4	0	0	0	8	0	0	0	6	0	0	0	19	0	0	0
22	Repair of supra-aortic stenosis	0	0	0	0	7	0	0	0	15	1 (6.7)	0	1 (6.7)	1	0	0	0	23	1 (4.3)	0	1 (4.3)
23	Repair of subaortic stenosis (including Redo)	0	0	0	0	3	0	0	0	38	0	0	0	1	0	0	0	42	0	0	0
24	Aortic valve plasty ± VSD Closure	10	0	0	0	10	0	0	0	20	0	0	0	5	0	0	0	45	0	0	0
25	Aortic valve replacement	0	0	0	0	2	0	0	0	17	0	0	0	37	0	0	1 (2.7)	56	0	0	1 (1.8)
26	AVR with annular enlargement	0	0	0	0	0	0	0	0	9	0	0	1 (11.1)	4	0	0	0	13	0	0	1 (7.7)
27	Aortic root Replace (except Ross)	0	0	0	0	0	0	0	0	13	0	0	0	14	0	0	0	27	0	0	0
28	Ross procedure	0	0	0	0	3	0	0	0	22	0	0	0					25	0	0	0
29	Bilateral pulmonary artery banding	179	8 (4.5)	0	19 (10.6)	11	1 (9.1)	0	2 (18.2)	0	0	0	0	0	0	0	0	190	9 (4.7)	0	21 (11.1)
Total		653	21 (3.2)	0	52 (8.0)	1520	19 (1.3)	1 (0.1)	44 (2.9)	1,328	8 (0.6)	0	15 (1.1)	328	3 (0.9)	0	4 (1.2)	3,829	51 (1.3)	1 (0.03)	115 (3.0)
(), % mortality																					
*SP* systemic-pulmonary; *PAB* pulmonary artery banding; *PA* pulmonary artery; *RVOT* right ventricular outflow tract; *CA* coronary artery; *AV fistula* arteriovenous fistula; *TCPC* total cavopulmonary connection; *AV valve* atrioventricular valve; *VSD* ventricular septal defect; *AVR* aortic valve replacement

**Table 2:** Acquired (total, (1) + (2) + (4) + (5) + (6) + (7) + isolated operations for arrhythmia in (3); 31,802

(1) Valvular heart disease (total; 17,805)																	
	Valve	Cases	Operation					30-Day mortality				Hospital mortality		Redo			
			Mechanical	Bioprosthesis	Repair	Unknown	with CABG	Hospital		After discharge				Cases	30-Day mortality		Hospital mortality
								Replace	Repair	Replace	Repair	Replace	Repair		Hospital	After discharge	
Isolated	A	7893	926	6572	125	270	1629	118 (1.6)	4(3.2)	3 (0.03)	0	198 (2.6)	5 (4.0)	652	29 (4.4)	1(0.2)	48 (7.4)
	M	5126	344	969	3870	33	517	49 (3.7)	27(0.7)	0	0	91 (6.9)	40 (1.1)	598	16 (2.7)	0	25 (4.2)
	T	255	4	58	191	2	42	4 (6.5)	5(2.6)	0	0	8 (12.9)	10 (5.2)	77	2 (2.6)	0	5 (6.5)
	P	20	0	18	0	2	0	0	0	0	0	0	0	16	0	0	0
A + M		929					145	28 (3.0)		0		58 (6.2)		146	8 (5.5)	0	15 (10.3)
	A		135	758	29	7											
	M		98	351	476	4											
A + T		289					35	3 (1.0)		0		11 (3.8)		63	0	0	2 (3.2)
	A		31	252	3	3											
	T		0	0	283	6											
M + T		2483					221	33 (1.3)		0		60 (2.4)		300	7 (2.3)	0	12 (4.0)
	M		172	755	1549	7											
	T		4	22	2442	15											
A + M + T		593					76	23 (3.9)		0		33 (5.6)		101	6 (5.9)	0	9 (8.9)
	A		63	514	8	8											
	M		39	289	265	0											
	T		1	10	582	0											
Others		217					7	3 (1.4)		0		3 (1.4)		40	2 (5.0)	0	2 (5.0)
Total		17,805					2672	261 (1.5)		3 (0.1)		462 (2.6)		1993	70 (3.5)	2(0.1)	118 (5.9)

TAVR	Cases	30-Day mortality
	15,019	179 (1.2)

(2) Ischemic heart disease (total, (A) + (B); 11,227)																						
(A) Isolated CABG (total; (a) + (b); 10,097)																						
(a-1) On-pump arrest CABG (total;2134)																						
	Primary, elective				Primary, emergent				Redo, elective					Redo, emergent				Artery only	Artery + SVG	SVG only	Others	Unclear
	Cases	30 day mortality		Hospital mortality	Cases	30 day mortality		Hospital mortality	Cases	30 day mortality			Hospital mortality	Cases	30 day mortality		Hospital mortality					
		Hospital	After discharge			Hospital	After discharge			Hospital	After discharge				Hospital	After discharge						
1VD	36	1 (2.8)	0	1 (2.8)	3	1 (33.3)	0	1 (33.3)	2	0	0		0	1	1 (100.0)	0	1 (100.0)	15	16	9	2	0
2VD	236	2 (0.8)	0	2 (0.8)	21	5 (23.8)	0	6 (28.6)	2	0	0		0	0	0	0	0	22	221	15	1	0
3VD	844	11 (1.3)	1 (0.12)	21 (2.5)	72	6 (8.3)	0	7 (9.7)	5	0	0		0	1	0	0	0	48	846	21	6	1
LMT	730	16 (2.2)	0	22 (3.0)	133	4 (3.0)	0	6 (4.5)	3	0	0		0	0	0	0	0	74	757	31	3	1
no info	27	0	0	1 (3.7)	15	2 (13.3)	0	4 (26.7)	1	0	0		0	2	0	0	0	8	23	10	2	2
Total	1873	30 (1.6)	1 (0.05)	47 (2.5)	244	18 (7.4)	0	24 (9.8)	13	0	0		0	4	1 (25.0)	0	1 (25.0)	167	1863	86	14	4
Kawasaki	6	0	0	0	0	0	0	0	2	0	0		0	0	0	0	0	3	4	1	0	0
On dialysis	217	8 (3.7)		14 (6.5)	32	6 (18.8)	0	7 (21.9)	2	0	0		0	1	1 (100.0)	0	1 (100.0)	14	221	16	0	1
(), % mortality																						
*CABG* coronary artery bypass grafting; *1VD* one-vessel disease; *2VD* two-vessel disease; *3VD* three-vessel disease; *LMT* left main trunk; *SVG* saphenous vein graft																						
LMT includes LMT alone or LMT with other branch diseases																						

(a-2) On-pump beating CABG (total;2038)																					
	Primary, elective				Primary, emergent				Redo, elective				Redo, emergent				Artery only	Artery + SVG	SVG only	Others	Unclear
	Cases	30 day mortality		Hospital mortality	Cases	30 day mortality		Hospital mortality	Cases	30 day mortality		Hospital mortality	Cases	30 day mortality		Hospital mortality					
		Hospital	After discharge			Hospital	After discharge			Hospital	After discharge			Hospital	After discharge						
1VD	27	3 (11.1)	0	4 (14.8)	10	1 (10.0)	0	1 (10.0)	6	1 (16.7)	0	1 (16.7)	2	0	0	0	14	17	14	0	0
2VD	233	4 (1.7)	1 (0.4)	6 (2.6)	25	1 (4.0)	0	3 (12.0)	3	0	0	0	0	0	0	0	62	182	15	2	0
3VD	735	11 (1.5)	0	15 (2.0)	111	9 (8.1)	0	11 (9.9)	1	1 (100.0)	0	1 (100.0)	1	0	0	0	80	738	17	8	5
LMT	625	13 (2.1)	1 (0.16)	25 (4.0)	197	22 (11.2)	0	30 (15.2)	13	0	0	1 (7.7)	2	1 (50.0)	0	1 (50.0)	116	667	48	4	2
no info	30	0	0	0	13	1 (7.7)	0	2 (15.4)	3	0	0	0	1	0	0	1 (100.0)	13	24	6	0	4
Total	1650	31 (1.9)	2 (0.12)	50 (3.0)	356	34 (9.6)	0	47 (13.2)	26	2 (7.7)	0	3 (11.5)	6	1 (16.7)	0	2 (33.3)	285	1628	100	14	11
Kawasaki	4	0	0	0 (0.0)	0	0	0	0	0	0	0	0	0	0	0	0	0	1	0	0	0
On dialysis	252	14 (5.6)	0	25 (9.9)	43	6 (14.0)	0	10 (23.3)	7	2 (28.6)	0	2 (28.6)	0	0	0	0	26	254	21	1	0
(), % mortality																					
*CABG* coronary artery bypass grafting; *1VD* one-vessel disease; *2VD* two-vessel disease; *3VD* three-vessel disease; *LMT* left main trunk; *SVG* saphenous vein graft																					
LMT includes LMT alone or LMT with other branch diseases																					

(b) Off-pump CABG (total; 5925)																					
(Including cases of planned off-pump CABG in which, during surgery, the change is made to an on-pump CABG or on-pump beating-heart procedure)																					
	Primary, elective				Primary, emergent				Redo, elective				Redo, emergent				Artery only	Artery + SVG	SVG only	Others	Unclear
	Cases	30 day mortality		Hospital mortality	Cases	30 day mortality		Hospital mortality	Cases	30 day mortality		Hospital mortality	Cases	30 day mortality		Hospital mortality					
		Hospital	After discharge			Hospital	After discharge			Hospital	After discharge			Hospital	After discharge						
1VD	301	1 (0.3)	0	1 (0.3)	16	1 (6.3)	0	1 (6.3)	8	0	0	0	5	0	0	0	255	48	21	1	5
2VD	844	11 (1.3)	0	14 (1.7)	67	0	0	1 (1.5)	6	0	0	0	1	0	0	0	343	553	14	0	8
3VD	2129	17 (0.8)	2 (0.1)	34 (1.6)	160	3 (1.9)	0	5 (3.1)	17	0	0	0	2	0	0	0	493	1731	31	20	33
LMT	1913	13 (0.7)	1 (0.1)	23 (1.2)	318	11 (3.5)	0	17 (5.3)	16	0	0	0	4	0	0	0	681	1505	45	7	13
no info	78	1 (1.3)	0	1 (1.3)	31	1 (3.2)	0	4 (12.9)	5	1 (20.0)	0	1 (20.0)	4	1 (25.0)	0	1 (25.0)	34	62	12	0	10
Total	5265	43 (0.8)	3 (0.1)	73 (1.4)	592	16 (2.7)	0	28 (4.7)	52	1 (1.9)	0	1 (1.9)	16	1 (6.3)	0	1 (6.3)	1806	3899	123	28	69
Kawasaki	13	0	0	0	2	0	0	0	0	0	0	0	0	0	0	0	12	3	0	0	0
On dialysis	531	20 (3.8)	0	27 (5.1)	56	1 (1.8)	0	5 (8.9)	10	1 (10.0)	0	1 (10.0)	3	0	0	0	164	404	25	0	7
(), % mortality																					
*CABG* coronary artery bypass grafting; *1VD* one-vessel disease; *2VD* two-vessel disease; *3VD* three-vessel disease; *LMT* left main trunk; *SVG* saphenous vein graft																					
LMT includes LMT alone or LMT with other branch diseases																					

(c) Cases of conversion, during surgery, from off-pump CABG to on-pump CABG or on- pump beating-heart CABG (these cases are also included in category (b))																
	Primary, elective				Primary, emergent				Redo, elective				Redo, emergent			
	Cases	30 day mortality		Hospital mortality	Cases	30 day mortality		Hospital mortality	Cases	30 day mortality		Hospital mortality	Cases	30 day mortality		Hospital mortality
		Hospital	After discharge			Hospital	After discharge			Hospital	After discharge			Hospital	After discharge	
Converted to arrest	14	1 (7.1)	0	1 (7.1)	2	0	0	0	0	0	0	0	1	0	0	0
Converted to beating	84	2 (2.4)	0	7 (8.3)	20	4 (20.0)	0	4 (20.0)	3	0	0	0	0	0	0	0
Total	98	3 (3.1)	1 (1.0)	8 (8.2)	22	4 (18.2)	0	4 (18.2)	3	0	0	0	1	0	0	0
On dialysis	15	3 (20.0)	0	5 (33.3)	3	1 (33.3)	0	1 (33.3)	0	0	0	0	1	0	0	0
(), % mortality																
*CABG* coronary artery bypass grafting																

(B) Operation for complications of MI (total; 1130)											
	Chronic				Acute				Concomitant operation		
	Cases	30-Day mortality		Hospital mortality	Cases	30-Day mortality		Hospital mortality			
		Hospital	After discharge			Hospital	After discharge		CABG	MVP	MVR
Infarctectomy or aneurysmectomy	84	3 (3.6)	0	4 (4.8)	28	7 (25.0)	0	9 (32.1)	62	15	2
VSP closure	75	12 (16.0)	0	18 (24.0)	276	73 (26.4)	0	96 (34.8)	90	3	6
Cardiac rupture	24	3 (12.5)	0	6 (25.0)	271	96 (35.4)	0	110 (40.6)	40	0	4
Mitral regurgitation											
(1) Papillary muscle rupture	25	2 (8.0)	0	2 (8.0)	59	9 (15.3)	0	14 (23.7)	31	13	71
(2) Ischemic	111	9 (8.1)	0	17 (15.3)	30	3 (10.0)	0	5 (16.7)	112	91	50
Others	67	4 (6.0)	0	5 (7.5)	80	24 (30.0)	0	32 (40.0)	51	4	6
Total	386	33 (8.5)	0	52 (13.5)	744	212 (28.5)	0	266 (35.8)	386	126	139
(), % mortality											
*MI* myocardial infarction; *CABG* coronary artery bypass grafting; *MVP* mitral valve repair; *MVR* mitral valve replacement; *VSP* ventricular septal perforation											
Acute, within 2 weeks from the onset of myocardial infarction											

(3) Operation for arrhythmia (total; 6478)											
	Cases	30-Day mortality		Hospital mortality	Concomitant operation						
					Isolated	Congenital	Valve	IHD	Others	Multiple combination	
		Hospital	After discharge							2 categories	3 categories
Maze	3201	54 (1.7)	1 (0.03)	89 (2.8)	296	168	2556	515	294	598	33
For WPW	1	0	0	0	0	0	0	1	0	0	0
For ventricular tachyarrhythmia	30	1 (3.3)	0	2 (6.7)	5	3	14	13	2	9	1
Others	3246	64 (2.0)	2 (0.06)	105 (3.2)	206	162	2531	600	410	609	48
Total	6478	119 (1.8)	3 (0.05)	196 (3.0)	507	333	5101	1129	706	1216	82
(), % mortality											
*WPW* Wolff-Parkinson-White syndrome; *IHD* ischemic heart disease											
Except for 170 isolated cases, all remaining 5164 cases are doubly allocated, one for this subgroup and the other for the subgroup corresponding to the concomitant operations											

(4) Operation for constrictive pericarditis (total; 177)								
	CPB ( +)				CPB ( −)			
	Cases	30-Day mortality		Hospital mortality	Cases	30-Day mortality		Hospital mortality
		Hospital	After discharge			Hospital	After discharge	
Total	111	13 (11.7)	0	25 (22.5)	66	1 (1.5)	0	4 (6.1)
(), % mortality								

(5) Cardiac tumor (total; 660)								
	Cases	30-Day mortality		Hospital mortality	Concomitant operation			
		Hospital	After discharge		AVR	MVR	CABG	Others
Benign tumor	587	4 (0.7)	0	6 (1.0)	31	9	41	154
(Cardiac myxoma)	409	1 (0.2)	0	2 (0.5)	10	2	17	96
Malignant tumor	73	1 (1.4)	1 (1.4)	2 (2.7)	1	3	1	9
(Primary)	52	0	1 (1.9)	0	1	2	1	9
(), % mortality								
*AVR* aortic valve replacement; *MVR* mitral valve replacement; *CABG* coronary artery bypass grafting								

(6) HOCM and DCM (total; 237)								
	Cases	30-Day mortality		Hospital mortality	Concomitant operation			
		Hospital	After discharge		AVR	MVR	MVP	CABG
Myectomy	108	2 (1.9)	0	2 (1.9)	36	12	26	8
Myotomy	4	1 (25.0)	0	1 (25.0)	1	2	0	1
No-resection	117	1 (0.9)	0	4 (3.4)	27	43	74	8
Volume reduction surgery of the left ventricle	8	1 (12.5)	0	1 (12.5)	0	0	1	4
Total	237	5 (2.1)	0	8 (3.4)	64	57	101	21
(), % mortality								
*HOCM* hypertrophic obstructive cardiomyopathy; *DCM* dilated cardiomyopathy; *AVR* aortic valve replacement; *MVR* mitral valve replacement; *MVP* mitral valve repair; *CABG* coronary artery bypass grafting								

(7) Other open-heart operation (total; 1189)				
	Cases	30-Day mortality		Hospital mortality
		Hospital	After discharge	
Open-heart operation	456	50 (11.0)	0	68 (14.9)
Non-open-heart operation	733	60 (8.2)	0	96 (13.1)
Total	1189	110 (9.3)	0	164 (13.8)
(), % mortality

**Table 3:** Thoracic aorta (total; 23,104)

(1) Dissection (total; 11,917)																						
Stanford type	Acute								Chronic								Concomitant operation					
	A				B				A				B									
Replaced site	Cases	30-Day mortality		Hospital mortality	Cases	30-Day mortality		Hospital mortality	Cases	30-Day mortality		Hospital mortality	Cases	30-Day mortality		Hospital mortality	AVP	AVR	MVP	MVR	CABG	Others
		Hospital	After discharge			Hospital	After discharge			Hospital	After discharge			Hospital	After discharge							
Ascending Ao	2085	172 (8.2)	1 (0.05)	217 (10.4)	2	0	0	0	190	3 (1.6)	0	9 (4.7)	3	1 (33.3)	0	1 (33.3)	34	135	17	14	97	30
Aortic Root	201	25 (12.4)	0	31 (15.4)	1	0	0	0	81	4 (4.9)	0	6 (7.4)	2	0	0	0	39	196	4	3	60	7
Arch	2300	143 (6.2)	4 (0.17)	204 (8.9)	21	4 (19.0)	0	4 (19.0)	388	13 (3.4)	0	19 (4.9)	178	3 (1.7)	0	4 (2.2)	85	130	17	7	115	24
Aortic root + asc. Ao. + Arch	221	22 (10.0)	0	27 (12.2)	1	0	0	0	44	3 (6.8)	1 (2.3)	3 (6.8)	10	1 (10.0)	0	1 (10.0)	32	154	1	2	46	3
Descending Ao	41	5 (12.2)	0	6 (14.6)	31	2 (6.5)	0	3 (9.7)	79	3 (3.8)	0	3 (3.8)	184	3 (1.6)	0	8 (4.3)	1	0	0	0	6	0
Thoracoabdominal	0	0	0	0	9	1 (11.1)	0	1 (11.1)	66	6 (9.1)	0	6 (9.1)	168	9 (5.4)	0	11 (6.5)	0	0	0	0	0	0
Simple TEVAR	96	9 (9.4)	0	11 (11.5)	485	33 (6.8)	2 (0.4)	38 (7.8)	274	4 (1.5)	0	7 (2.6)	1176	11 (0.9)	0	13 (1.1)	0	0	0	0	0	2
Open SG with BR	1635	125 (7.6)	1 (0.06)	162 (9.9)	68	7 (10.3)	0	10 (14.7)	201	7 (3.5)	0	9 (4.5)	276	7 (2.5)	0	11 (4.0)	77	167	8	2	112	14
Open SG without BR	511	40 (7.8)	1 (0.20)	56 (11.0)	28	5 (17.9)	0	6 (21.4)	73	2 (2.7)	0	3 (4.1)	84	5 (6.0)	0	6 (7.1)	23	50	4	0	22	5
Arch TEVAR with BR	18	1 (5.6)	1 (5.56)	2 (11.1)	115	8 (7.0)	0	9 (7.8)	90	0	0	0	373	3 (0.8)	1 (0.3)	5 (1.3)	1	0	0	0	0	0
Thoracoabdominal TEVAR with BR	0	0	0	0	3	0	0	1 (33.3)	5	0	0	0	25	1 (4.0)	0	1 (4.0)	0	0	0	0	0	0
Other	12	4 (33.3)	0	4 (33.3)	20	3 (15.0)	0	4 (20.0)	8	0	0	0	35	1 (2.9)	0	1 (2.9)	0	0	1	0	0	0
Total	7120	546 (7.7)	8 (0.11)	720 (10.1)	784	63 (8.0)	2 (0.3)	76 (9.7)	1499	45 (3.0)	1 (0.1)	65 (4.3)	2514	45 (1.8)	1 (0.0)	62 (2.5)	292	832	52	28	458	85
(), % mortality																						
Ao aorta; AVP aortic valve repair; AVR aortic valve replacement; MVP mitral valve repair; MVR mitral valve replacement; CABG coronary artery bypass grafting; TEVAR thoracic endovascular aortic (aneurysm) repair																						
Acute, within 2 weeks from the onset																						

(2) Non-dissection (total; 11,907)														
Replaced site	Unruptured				Ruptured				Concomitant operation					
	Cases	30-Day mortality		Hospital mortality	Cases	30-Day mortality		Hospital mortality	AVP	AVR	MVP	MVR	CABG	Others
		Hospital	After discharge			Hospital	After discharge							
Ascending Ao	1373	15 (1.1)	2 (0.15)	37 (2.7)	68	8 (11.8)	0	11 (16.2)	40	957	48	52	136	89
Aortic Root	1135	21 (1.9)	2 (0.18)	34 (3.0)	71	8 (11.3)	0	10 (14.1)	300	807	60	37	133	82
Arch	2038	38 (1.9)	0	67 (3.3)	95	17 (17.9)	0	21 (22.1)	27	615	33	28	233	58
Aortic root + asc. Ao. + Arch	319	7 (2.2)	0	10 (3.1)	11	2 (18.2)	0	2 (18.2)	47	246	8	4	28	10
Descending Ao	305	13 (4.3)	1 (0.33)	22 (7.2)	37	6 (16.2)	0	11 (29.7)	0	12	0	0	16	1
Thoracoabdominal	357	11 (3.1)	0	22 (6.2)	47	5 (10.6)	0	8 (17.0)	0	0	0	0	1	0
Simple TEVAR	2392	31 (1.3)	7 (0.29)	62 (2.6)	403	52 (12.9)	1 (0.25)	75 (18.6)	0	0	1	0	3	3
Open SG with BR	1252	31 (2.5)	0	54 (4.3)	84	13 (15.5)	0	17 (20.2)	23	156	14	3	166	19
Open SG without BR	441	9 (2.0)	0	22 (5.0)	35	6 (17.1)	0	7 (20.0)	12	87	7	3	49	8
Arch TEVAR with BR	1101	18 (1.6)	3	36 (3.3)	76	13 (17.1)	0	20 (26.3)	0	1	0	1	3	0
Thoracoabdominal TEVAR with BR	111	9 (8.1)	0	13 (11.7)	7	1 (14.3)	0	2 (28.6)	0	0	0	0	1	0
Other	127	2 (1.6)	0	3 (2.4)	22	2 (9.1)	0	3 (13.6)	0	5	2	1	9	4
Total	10,951	205 (1.9)	15 (0.14)	382 (3.5)	956	133 (13.9)	1 (0.10)	187 (19.6)	449	2886	173	129	778	274
(), % mortality														
*Ao* aorta; *AVP* aortic valve repair; *AVR* aortic valve replacement; *MVP* mitral valve repair; *MVR* mitral valve replacement; *CABG* coronary artery bypass grafting; *TEVAR* thoracic endovascular aortic(aneurysm) repair

**Table 4:** Pulmonary thromboembolism (total; 172)

	Cases	30-Day mortality		Hospital mortality
		Hospital	After discharge	
Acute	118	22 (18.6)	0	26 (22.0)
Chronic	54	2 (3.7)	0	2 (3.7)
Total	172	24 (14.0)	0	28 (16.3)

(), Mortality %

**Table 5:** Implantation of VAD (total; 150)

	Cases	30-Day mortality		Hospital mortalitys
		Hospital	After discharge	
Implantation of VAD	150	1 (0.7)	0	7 (4.7)

(), Mortality %

*VAD* ventricular assist devise

**Table 6:** Heart transplantation (total; 115)

	Cases	Hospital mortality
Heart transplantation	115	2 (1.7)
Heart and lung transplantation	0	0
Total	115	2 (1.7)

(), Mortality %

Among the 8084 procedures for congenital heart disease conducted in 2023, 6190 were open-heart surgeries, with an overall hospital mortality rate of 1.7% (Table 1). The number of surgeries for neonates and infants in 2023 significantly decreased compared to that in 2013 (3730 vs. 4954); on the other hand, hospital mortality did not significantly differ compared to those in 2013 (6.3% vs. 6.0% for neonates and 2.9% vs. 2.4% for infants) despite the increasing ratio of surgeries for severe cases. In 2023, atrial septal defect (1290 cases) and ventricular septal defect (1312 cases) were the most common diseases as previously reported, with patients aged ≥ 18 years accounting for 38% of atrial septal defect and ventricular septal defect surgeries [7].

Hospital mortality of open heart surgeries for complex congenital heart disease within the past 10 years was as follows (2013 [7], 2018 [8], and 2023): complete atrioventricular septal defect (0.6%, 2.5%, and 0.5%); tetralogy of Fallot (1.4%, 1.1%, and 0.6%); transposition of the great arteries with the intact septum (3.6%, 2.1%, and 5.1%), ventricular septal defect (5.2%, 6.9%, and 4.3%), single ventricle (5.7%, 5.1%, and 4.5%); and hypoplastic left heart syndrome (9.1%, 8.8%, and 7.3%). Currently, right heart bypass surgery has been commonly performed (285 bidirectional Glenn procedures, excluding 26 Damus–Kaye–Stansel procedures, and 298 Fontan type procedures, including total cavopulmonary connection) with acceptable hospital mortality rates (1.1% and 1.7%). The Norwood type I procedure was performed in 117 cases, with a relatively low hospital mortality rate (9.4%) (Table 1).

Valvular heart disease procedures were performed more than that in the previous year. Isolated aortic valve replacement/repair with/without coronary artery bypass grafting (CABG) (*n* = 7893) was 0.8% more than that in the previous year (*n* = 7834) and 25.4% fewer than that 5 years ago (*n* = 10,584 in 2018), as opposed to the rapid increase of transcatheter aortic valve replacement (*n* = 13,534 and 15,019 in 2022 and 2023). Isolated mitral valve replacement/repairs with/without CABG (*n* = 5126) was 8.9% more than that in the previous year (*n* = 4708) and 4.6% more than that 5 years ago (*n* = 4898 in 2018). Aortic and mitral valve replacement with bioprosthesis were performed in 8096 and 2364 cases, respectively. The rate at which bioprosthesis was used had dramatically increased from 30% in the early 2000s [9, 10] to 87.5% and 78.4% in 2023 for aortic and mitral positions, respectively. Additionally, CABG was performed concurrently in 15.0% of all valvular procedures (17.8% in 2013 [7] and 17.3% in 2018 [8]). Valve repair was common in mitral and tricuspid valve positions (6160 and 3498 cases, respectively) but less common in aortic valve positions (165 patients, only 1.7% of all aortic valve procedures). Mitral valve repair accounted for 67.5% of all mitral valve procedures. Hospital mortality rates for isolated valve replacement for aortic and mitral positions were 2.6% and 6.9%, respectively, but only 1.1% for mitral valve repair. Moreover, hospital mortality rates for redo-isolated valve surgery for the aortic and mitral positions were 7.4% and 4.2%, respectively. Finally, overall hospital mortality rates did not significantly improve over the past 10 years (3.1% in 2013 [7], 3.5% in 2018 [8], and 2.6% in 2023) (Table 2).

Isolated CABG had been performed in 10,097 cases, accounting for only 65.9% of the procedures performed 10 years ago (*n* = 15,333 in 2013) [7]. Of the aforementioned cases, 5925 (58.7%) underwent off-pump CABG, with a success rate of 97.9%. The percentage of planned off-pump CABG in 2023 was similar to that in 2022. Hospital mortality associated with primary elective CABG procedures among 8788 cases accounted for 1.9%, which is slightly higher than that in 2013 (1.7%) [7]. Hospital mortality for primary emergency CABG among 1192 cases remained high (8.3%). The percentage of conversion from off-pump to on-pump CABG or on-pump beating-heart CABG was 1.9% among the primary elective CABG cases, with a hospital mortality rate of 8.2%. Patients with end-stage renal failure on dialysis had higher hospital mortality rates than overall mortality, regardless of surgical procedure (on-pump arrest, on-pump beating, and off-pump). This study excluded concomitant CABGs alongside other major procedures under the ischemic heart disease category but rather under other categories, such as valvular heart disease and thoracic aortic aneurysm. Accordingly, the overall number of CABGs in 2023, including concomitant CABG with other major procedures, was 14,454 (Table 2).

Arrhythmia management was primarily or concomitantly performed in 6478 cases, with a hospital mortality rate of 3.0%. Pacemaker and implantable cardioverter-defibrillator implantation were not included in this category (Table 2).

In 2023, 23,104 procedures for thoracic and thoracoabdominal aortic diseases were performed, among which aortic dissection and non-dissection accounted for 11,917 and 11,907, respectively. The number of surgeries for aortic dissection this year was 4.2% higher than that in the preceding year (*n* = 11,438 in 2022). Hospital mortality rates for the 7120 Stanford type A acute aortic dissections remained high (10.1%). The number of procedures for non-aortic dissections increased by 6.7%, with a hospital mortality rate of 4.8% for all aneurysms and 3.5% and 19.6% for unruptured and ruptured aneurysms, respectively. Thoracic endovascular aortic repair (TEVAR) has been performed for aortic diseases at an increasing rate [2–5]. Stent graft placement was performed in 5536 patients with aortic dissection, including 2660 TEVARs and 2876 open stent graftings. Moreover, 1,574 and 360 cases underwent TEVAR and open stent grafting for type B chronic aortic dissection, accounting for 59.2% and 12.5% of the total number of cases, respectively. Hospital mortality rates associated with simple TEVAR for type B aortic dissection were 7.8% and 1.1% for acute and chronic cases, respectively. Stent graft placement was performed in 5902 patients with non-dissected aortic aneurysms, among which 4,090 were TEVARs (a 3.8% increase compared to that in 2022, *n* = 3942) and 1812 were open stent graftings (a 13.6% increase compared to that in 2022, *n* = 1595). Hospital mortality rates were 3.1% and 20.0% for TEVARs and 4.5% and 20.2% for open stenting in unruptured and ruptured aneurysms, respectively (Table 3).

### (B) General thoracic surgery

**Fig. 2:**
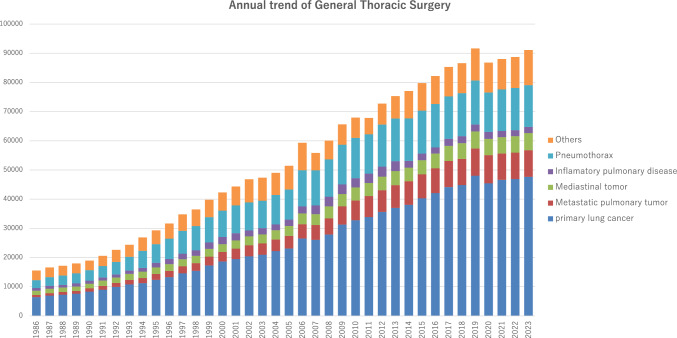
Annual trend of general thoracic surgery

The 2023 survey of general thoracic surgeries comprised 693 surgical units, with bulk data submitted via a web-based collection system established by the NCD [4]. General thoracic surgery departments reported 91,087 procedures in 2023 (Table 7), which is 2.2 times more than that in 2000 and 4498 more procedures than in 2018 [8] (Fig. 2). It increased compared to that in 2020 (the first year of the COVID-19 pandemic: 86,813) [3] by 4.9% and recovered the level of 2019 (before the COVID-19 pandemic: 91,626) [2].

**Table 7:** Total cases of general thoracic surgery during 2022

	Cases	%
Benign pulmonary tumor	2454	2.7
Primary lung cancer	47,659	52.3
Other primary malignant pulmonary tumor	423	0.5
Metastatic pulmonary tumor	9140	10.0
Tracheal tumor	90	0.1
Pleural tumor including mesothelioma	570	0.6
Chest wall tumor	648	0.7
Mediastinal tumor	5851	6.4
Thymectomy for MG without thymoma	97	0.1
Inflammatory pulmonary disease	2122	2.3
Empyema	4384	4.8
Bullous disease excluding pneumothorax	281	0.3
Pneumothorax	14,311	15.7
Chest wall deformity	297	0.3
Diaphragmatic hernia including traumatic	44	0.0
Chest trauma excluding diaphragmatic hernia	578	0.6
Lung transplantation	127	0.1
Others	2011	2.2
Total	91,087	100.0

In 2023, 47,659 procedures for primary lung cancer had been performed which increased by 1.6% compared to that of 2022 (46,888) [5], and recovered the level of 2019 (48,052) [2], similar to the total number of surgeries in general thoracic surgery. The procedures for lung cancer account for 52% of all general thoracic surgeries in 2023.

Information about the number of video-assisted thoracoscopic surgery (VATS), which is defined as surgical procedures using a skin incision less than 8 cm including a mini-thoracotomy (hybrid) approach, has been available since the 2015 annual report. Tables 8, 9, 11, 14, 15, 16, 18, 19, 20, 21, 22, 24, 25, and 26 present the number of VATS procedures for benign pulmonary tumors, primary lung cancer, metastatic pulmonary tumor, chest wall tumor, mediastinal tumor, thymectomy for myasthenia gravis, inflammatory pulmonary disease, empyema, descending necrotizing mediastinitis, bullous diseases, pneumothorax, diaphragmatic hernia, chest trauma and other respiratory surgeries in 2023, respectively.

A total of 2454 procedures for benign pulmonary tumors had been conducted in 2023 (Table 8). Hamartomas were the most frequent benign pulmonary tumors diagnosed, with 2312 patients (94%) undergoing VATS.

**Table 8:** Benign pulmonary tumor

	Cases	30-Day mortality		Hospital mortality	By VATS
		Hospital	After discharge		
1. Benign pulmonary tumor					
Hamartoma	475	0	0	0	461
Sclerosing hemangioma	89	0	0	0	86
Papilloma	28	1 (3.6)	0	1 (3.6)	27
Mucous gland adenoma bronchial	27	0	0	0	26
Fibroma	133	0	1 (0.8)	0	123
Lipoma	9	0	0	0	8
Neurogenic tumor	9	0	0	0	7
Clear cell tumor	0	0	0	0	0
Leiomyoma	13	0	0	0	12
Chondroma	9	0	0	0	9
Inflammatory myofibroblastic tumor	3	0	0	0	3
Pseudolymphoma	24	0	0	0	23
Histiocytosis	9	0	0	0	9
Teratoma	6	0	0	0	3
Others	1620	1 (0.1)	1 (0.1)	2 (0.1)	1515
Total	2454	2 (0.08)	2 (0.08)	3 (0.12)	2312

(), Mortality %

Tables 9 and 10 show additional information on primary malignant pulmonary tumors. Accordingly, the most frequently diagnosed lung cancer subtype was adenocarcinoma (71% of all lung cancers), followed by squamous cell carcinoma (17%). Sublobar resection was performed in 18,891 lung cancer cases (40% of all cases) and lobectomy in 28,371 cases (60% of all cases). Sleeve lobectomy was performed in 311 cases (0.7% of all cases), while pneumonectomy was required in 126 cases (0.3% of all cases). VATS lobectomy was performed in 18,403 cases of lung cancer (65% of all lobectomy cases). RATS lobectomy was performed in 5256 cases of lung cancer (19% of all lobectomy cases). Patients aged ≥ 80 years who underwent lung cancer surgery accounted for 8150 (17%). Among those who died within 30 days postoperatively, 101 and 51 died before and after hospital discharge, respectively. Overall, 152 patients died within 30 days postoperatively (30-day mortality rate, 0.3%), while 211 died before discharge (hospital mortality rate, 0.4%). Moreover, 30-day mortality rates according to the procedure were 0.1%, 0.4%, and 5.6% for segmentectomy, lobectomy, and pneumonectomy, respectively. Interstitial pneumonia had been the leading cause of death after lung cancer surgery, followed by pneumonia, cardiovascular events, respiratory failure, bronchopleural fistule, and brain infarction or bleeding.

**Table 9:** Primary malignant pulmonary tumor

	Cases	30-Day mortality			Hospital mortality	VATS	Robotic surgery
		Hospital	After discharge				
2. Primary malignant pulmonary tumor	48,082	102 (0.2)	51 (0.1)		215 (0.4)	34,655	6900
Lung cancer	47,659	101 (0.2)	51 (0.1)		211 (0.4)	34,655	6900
Histological classification							
Adenocarcinoma	33,821	43 (0.1)	22 (0.07)		81 (0.2)		
Squamous cell carcinoma	8152	40 (0.5)	18 (0.2)		94 (1.2)		
Large cell carcinoma	314	0	2 (0.6)		2 (0.6)		
LCNEC	530	4 (0.8)	0		9 (1.7)		
Small cell carcinoma	843	0	1 (0.1)		2 (0.2)		
Adenosquamous carcinoma	549	1 (0.2)	1	(0.2)	4 (0.7)		
Carcinoma with pleomorphic, sarcomatoid or sarcomatous elements	531	7 (1.3)	5 (0.9)		12 (2.3)		
Carcinoid	279	0	0		0		
Carcinomas of salivary-gland type	42	0	0		0		
Unclassified	40	1 (2.5)	0		1 (2.5)		
Multiple lung cancer	2145	4 (0.2)	1 (0.0)		5 (0.2)		
Others	378	1 (0.3)	1 (0.3)		1 (0.3)		
Operative procedure							
Wedge resection	9464	5 (0.1)	8 (0.1)		17 (0.2)	8872	36
Segmental excision	9427	8 (0.1)	5 (0.05)		18 (0.2)	7190	1596
(Sleeve segmental excision)	19	0	0		0	11	1
Lobectomy	28,371	81 (0.3)	36 (0.13)		166 (0.6)	18,403	5256
(Sleeve lobectomy)	311	2 (0.6)	1 (0.3)		5 (1.6)	48	18
Pneumonectomy	126	7 (5.6)	0		8 (6.3)	8	2
(Sleeve pneumonectomy)	4	1 (25.0)	0		1 (25.0)	0	0
Other bronchoplasty	35	0	0		0	3	2
Pleuropneumonectomy	1	0	0		0	0	0
Others	194	0	2 (1.0)		2 (1.0)	145	4
Multiple incision for multiple lung cancer	41	0	0		0	34	4
Sarcoma	39	0	0		2 (5.1)		
AAH	125	0	0		1 (0.8)		
Lymphoma	180	1 (0.6)	0		1 (0.6)		
Others	79	0	0		0		

(), Mortality %

**Table 10:** Details of lung cancer operations

TNM	
c-Stage	Cases
0	2203
IA1	9404
IA2	14,200
IA3	8227
IB	5022
IIA	1655
IIB	3607
IIIA	2307
IIIB	372
IIIC	18
IVA	375
IVB	113
NA	120
Total	47,623

Sex	Cases
Male	28,374
Female	19,249
NA	0
Total	47,623

Cause of death	Cases
Cardiovascular	39
Pneumonia	78
Pyothorax	3
Bronchopleural fistula	16
Respiratory failure	29
Pulmonary embolism	3
Interstitial pneumonia	101
Brain infarction or bleeding	15
Others	116
Unknown	35
Total	435

p-Stage	Cases
0 (pCR)	3154
IA1	9714
IA2	11,337
IA3	5520
IB	6713
IIA	1292
IIB	4334
IIIA	3534
IIIB	685
IIIC	11
IVA	878
IVB	93
NA	358
Total	47,623

Age (y)	Cases
< 20	21
20–29	65
30–39	201
40–49	1069
50–59	3795
60–69	10,424
70–79	23,897
80–89	8006
≥ 90	144
NA	1
Total	47,623

The procedures for metastatic pulmonary tumors performed in 2023 (9140) were similar to those in 2022 (9055) [5] (Table 11). Among such procedures, the most frequent primary tumor was colorectal cancer (47% of all cases).

**Table 11:** Metastatic pulmonary tumor

	Cases	30-Day mortality		Hospital mortality	VATS	Robotic surgery
		Hospital	After discharge			
3. Metastatic pulmonary tumor	9140	7 (0.1)	5 (0.05)	11 (0.12)	8409	512
Colorectal	4305	2 (0.05)	1 (0.02)	4 (0.09)	3982	238
Hepatobiliary/Pancreatic	582	0	1 (0.2)	0	553	41
Uterine	544	1 (0.2)	0	1 (0.18)	497	35
Mammary	574	0	0	0	550	36
Ovarian	74	0	0	0	65	4
Testicular	50	0	0	0	43	4
Renal	792	0	0	0	738	42
Skeletal	91	0	0	0	75	3
Soft tissue	237	0	0	0	196	7
Otorhinolaryngological	464	1 (0.2)	1 (0.2)	2 (0.43)	438	32
Pulmonary	439	1 (0.2)	0	1 (0.23)	353	15
Others	988	2 (0.2)	2 (0.2)	3 (0.30)	919	55

(), Mortality %

A total of 90 procedures for tracheal tumors, including 32, 31, and 27 cases of primary malignant, metastatic, and benign tracheal tumors, respectively, were performed in 2023. Further, 15 patients underwent sleeve resection and reconstruction (Table 12).

**Table 12:** Tracheal tumor

	Cases	30-Day mortality		Hospital mortality
		Hospital	After discharge	
4. Tracheal tumor	90	1 (1.1)	0	1 (1.1)
A. Primary malignant tumor				
Histological classification				
Squamous cell carcinoma	11	0	0	0
Adenoid cystic carcinoma	7	0	0	0
Mucoepidermoid carcinoma	6	0	0	0
Others	8	0	0	0
Total	32	0	0	0
B. Metastatic/invasive malignant tumor e.g. invasion of thyroid cancer				
	31	1 (3.2)	0	1 (3.2)
C. Benign tracheal tumor				
Papilloma	2	0	0	0
Adenoma	1	0	0	0
Neurofibroma	1	0	0	0
Chondroma	0	0	0	0
Leiomyoma	6	0	0	0
Others	17	0	0	0
Histology unknown	0	0	0	0
Total	27	0	0	0
Operative procedure				
Sleeve resection with reconstruction	15	0	0	0
Wedge with simple closure	1	0	0	0
Wedge with patch closure	0	0	0	0
Total laryngectomy with tracheostomy	0	0	0	0
Others	3	0	0	0
Unknown	0	0	0	0
Total	19	0	0	0

(), Mortality %

Overall, 570 pleural tumors had been diagnosed in 2023 (Table 13), with diffuse malignant pleural mesothelioma as the most frequent histologic diagnosis. Total pleurectomy was performed in 108 cases and extrapleural pneumonectomy in 14 cases. The 30-day mortality rate was 0% after total pleurectomy and extrapleural pneumonectomy, respectively.

**Table 13:** Tumor of pleural origin

5. Tumor of pleural origin				
Histological classification	Cases	30-Day mortality		Hospital mortality
		Hospital	After discharge	
Solitary fibrous tumor	122	0	0	0
Diffuse malignant pleural mesothelioma	186	2 (1.1)	1 (0.5)	4 (2.2)
Localized malignant pleural mesothelioma	33	1 (3.0)	0	1 (3.0)
Others	229	1 (0.4)	2 (0.9)	3 (1.3)
Total	570	4 (0.7)	3 (0.5)	8 (1.4)

Operative procedure	Cases	30-Day mortality		Hospital mortality
		Hospital	After discharge	
Extrapleural pneumonectomy	14	0	0	0
Total pleurectomy	108	0	0	0
Others	64	2 (3.1)	1 (1.6)	4 (6.3)
Total	186	2 (1.1)	1 (0.5)	4 (2.2)

(), Mortality %

Overall, 648 chest wall tumor resections were performed in 2023, including 136, 192, and 320 cases of primary malignant, metastatic, and benign tumors, respectively (Table 14).

**Table 14:** Chest wall tumor

6. Chest wall tumor					
	Cases	30-Day mortality		Hospital mortality	VATS
		Hospital	After discharge		
Primary malignant tumor	136	2 (1.5)	0	2 (1.5)	42
Metastatic malignant tumor	192	0	0	1 (0.5)	69
Benign tumor	320	0	0	0	251
Total	648	2 (0.3)	0	3 (0.5)	362

(), Mortality %

In 2023, 5851 mediastinal tumors were resected, which increased by 1.0% that in 2022 (5652) (Table 15) [5]. Thymic epithelial tumors, including 2210 thymomas, 381 thymic carcinomas, and 55 thymic carcinoids, were the most frequently diagnosed mediastinal tumor subtype in 2023.

**Table 15:** Mediastinal tumor

	Cases	30-Day mortality		Hospital mortality	By VATS	Robotic surgery
		Hospital	After discharge			
7. Mediastinal tumor	5851	8 (0.14)	3 (0.05)	13 (0.2)	4624	1864
Thymoma*	2210	2 (0.1)	1 (0.0)	2 (0.1)	1632	769
Thymic cancer	381	1 (0.3)	0	1 (0.3)	226	107
Thymus carcinoid	55	1 (1.8)	0	1 (1.8)	32	11
Germ cell tumor	80	0	0	1 (1.3)	42	21
Benign	49	0	0	0	34	16
Malignant	31	0	0	1 (3.2)	8	5
Neurogenic tumor	517	1 (0.2)	0	1 (0.2)	481	165
Congenital cyst	1319	0	1 (0.1)	2 (0.2)	1257	479
Goiter	93	0	0	0	45	9
Lymphatic tumor	186	1 (0.5)	1 (0.5)	2 (1.1)	133	40
Excision of pleural recurrence of thymoma	63	0	0	0	43	8
Thymolipoma	23	0	0	0	17	5
Others	924	2 (0.2)	0	3 (0.3)	716	250

(), Mortality %

A total of 477 patients underwent thymectomy for myasthenia gravis (Table 16), among which 380 procedures (80%) were associated with thymoma in 2023.

**Table 16:** Thymectomy for myasthenia gravis

	*Cases*	30-Day mortality		Hospital mortality	By VATS	Robotic surgery
		Hospital	After discharge			
8. Thymectomy for myasthenia gravis	477	2 (0.4)	0	2 (0.4)	333	158
With thymoma	380	2 (0.5)	0	2 (0.5)	257	129

(), Mortality %

Overall, 24,024 patients underwent procedures for non-neoplastic disease in 2023. Accordingly, 2122 patients underwent lung resection for inflammatory lung diseases (Tables 17, 18), among which 412 and 251 patients were associated with mycobacterial and fungal infections, respectively. Procedures for inflammatory pseudotumor were performed in 942 cases (44%).

**Table 17:** Operations for non-neoplastic diseases: A + B + C + D + E + F + G + H + I

	Cases	30-Day mortality		Hospital mortality
		Hospital	After discharge	
9. Operations for non-neoplastic diseases	24,024	293 (1.2)	39 (0.2)	576 (2.4)

**Table 18:** A. Inflammatory pulmonary disease

	Cases	30-Day mortality		Hospital mortality	VATS
		Hospital	After discharge		
A. Inflammatory pulmonary disease	2122	8 (0.4)	1 (0.0)	13 (0.6)	1662
Tuberculous infection	29	1 (3.4)	0	1 (3.4)	25
Mycobacterial infection	412	0	0	0	370
Fungal infection	251	2 (0.8)	0	3 (1.2)	178
Bronchiectasis	58	0	0	1 (1.7)	42
Tuberculous nodule	39	0	0	0	37
Inflammatory pseudotumor	942	0	0	1 (0.1)	883
Interpulmonary lymph node	65	0	0	0	63
Others	326	5 (1.5)	1 (0.3)	7 (2.1)	64

(), Mortality %

A total of 4384 procedures were performed for empyema (Table 19), among which 3698 (84%) were acute and 686 (16%) were chronic. Further, pleural fistulas developed in 544 and 276 patients with acute and chronic empyema, respectively. The hospital mortality rate was 14.2% among patients with acute empyema with fistula.

**Table 19:** B. Empyema

	Cases	30-Day mortality		Hospital mortality	by VATS
		Hospital	After discharge		
Acute empyema	3698	90 (2.4)	7 (0.2)	197 (5.3)	3195
With fistula	544	29 (5.3)	0	77 (14.2)	296
Without fistula	3108	60 (1.9)	7 (0.2)	118 (3.8)	2858
Unknown	46	1 (2.2)	0	2 (4.3)	41
Chronic empyema	686	19 (2.8)	2 (0.3)	63 (9.2)	382
With fistula	276	10 (3.6)	0	34 (12.3)	83
Without fistula	359	8 (2.2)	0	26 (7.2)	255
Unknown	51	1 (2.0)	2 (3.9)	3 (5.9)	44
Total	4384	109 (2.5)	9 (0.2)	260 (5.9)	3577

(), Mortality %

Further, 134 operations were performed for descending necrotizing mediastinitis (Table 20), with a hospital mortality rate of 6.7%.

**Table 20:** C. Descending necrotizing mediastinitis

	Cases	30-Day mortality		Hospital mortality	VATS
		Hospital	After discharge		
C. Descending necrotizing mediastinitis	134	6 (4.5)	0	9 (6.7)	105

(), Mortality %

A total of 281 procedures were conducted for bullous diseases (Table 21), while 19 patients underwent lung volume reduction surgery.

**Table 21:** D. Bullous diseases

	Cases	30-Day mortality		Hospital mortality	VATS
		Hospital	After discharge		
D. Bullous diseases	281	0	0	2 (0.7)	256
Emphysematous bulla	200	0	0	0	186
Bronchogenic cyst	14	0	0	2 (14.3)	12
Emphysema with LVRS	19	0	0	0	19
Others	48	0	0	0	39

(), Mortality %

*LVRS* lung volume reduction surgery

A total of 14,311 procedures were performed for pneumothorax (Table 22). Among the 9995 procedures for spontaneous pneumothorax, 2291 (23%) were bullectomies alone, while 7019 (70%) required additional procedures, such as coverage with artificial material, as well as parietal pleurectomy. A total of 4316 procedures for secondary pneumothorax were performed, with chronic obstructive pulmonary disease (COPD) being the most prevalent associated disease (2576 cases, 60%). The hospital mortality rate for secondary pneumothorax associated with COPD was 1.4%.

**Table 22:** E. Pneumothorax

Cases	30-Day mortality		Hospital mortality	VATS
	Hospital	After discharge		
14,311	80 (0.6)	22 (0.2)	141 (1.0)	13,897

Spontaneous pneumothorax					
Operative procedure	Cases	30-Day mortality		Hospital mortality	VATS
		Hospital	After discharge		
Bullectomy	2291	4 (0.2)	1 (0.0)	8 (0.3)	2249
Bullectomy with additional procedure	7019	7 (0.1)	3 (0.04)	13 (0.2)	6917
Coverage with artificial material	6824	7 (0.1)	3 (0.04)	12 (0.2)	6725
Parietal pleurectomy	40	0	0	0	38
Coverage and parietal pleurectomy	48	0	0	0	48
Others	107	0	0	1 (0.9)	106
Others	681	3 (0.4)	0	5 (0.7)	641
Unknown	4	0	0	0	4
Total	9995	14 (0.1)	4 (0.0)	26 (0.3)	9811

Secondary pneumothorax					
Associated disease	Cases	30-Day mortality		Hospital mortality	VATS
		Hospital	After discharge		
COPD	2998	24 (0.8)	11 (0.4)	42 (1.4)	2851
Tumorous disease	150	5 (3.3)	2 (1.3)	9 (6.0)	137
Catamenial	206	0	0	1 (0.5)	204
LAM	40	0	0	0	40
Others (excluding pneumothorax by trauma)	922	37 (4.0)	5 (0.5)	63 (6.8)	854
Unknown	0	0	0	0	0

Operative procedure	Cases	30 Day mortality		Hospital mortality	VATS
		Hospital	After discharge		
Bullectomy	781	6 (0.8)	0	13 (1.7)	766
Bullectomy with additional procedure	2576	29 (1.1)	7 (0.3)	47 (1.8)	2,495
Coverage with artificial material	2489	28 (1.1)	6 (0.2)	46 (1.8)	2411
Parietal pleurectomy	3	0	0	0	3
Coverage and parietal pleurectomy	18	0	0	0	18
Others	66	1 (1.5)	1 (1.5)	1 (1.5)	63
Others	947	29 (3.1)	11 (1.2)	53 (5.6)	817
Unknown	12	2 (16.7)	0	2 (16.7)	8
Total	4316	66 (1.5)	18 (0.4)	115 (2.7)	4086

(), Mortality %

The 2023 survey reported 297 procedures for chest wall deformity (Table 23). However, this may have been underestimated because the Nuss procedure for pectus excavatum was more likely performed in pediatric surgery centers not associated with the Japanese Association for Thoracic Surgery.

**Table 23:** F. Chest wall deformity

	Cases	30-Day mortality		Hospital mortality
		Hospital	After discharge	
F. Chest wall deformity	297	1 (0.3)	0	0
Funnel chest	292	0	0	0
Others	5	1 (20.0)	0	2 (40.0)

(), Mortality %

Surgical treatment for diaphragmatic hernia was performed in 44 patients (Table 24). This may have been underestimated because procedures may have been classified as gastrointestinal surgery.

**Table 24:** G. Diaphragmatic hernia

	Cases	30-Day mortality		Hospital mortality	VATS
		Hospital	After discharge		
G. Diaphragmatic hernia	44	1 (2.3)	0	1 (2.3)	22
Congenital	7	0	0	0	3
Traumatic	7	0	0	0	2
Others	30	1 (3.3)	0	1 (3.3)	17

(), Mortality %

The survey reported 578 procedures for chest trauma, excluding iatrogenic injuries (Table 25), with a hospital mortality rate of 9.2%.

**Table 25:** H. Chest trauma

	Cases	30-Day mortality		Hospital mortality	VATS
		Hospital	After discharge		
H. Chest trauma	578	40 (6.9)	0	53 (9.2)	318

(), Mortality %

Table 26 summarizes the procedures for other diseases, including 110 and 124 cases of arteriovenous malformation and pulmonary sequestration, respectively.

**Table 26:** I. Other respiratory surgery

	Cases	30-Day mortality		Hospital mortality	VATS
		Hospital	After discharge		
I. Other respiratory surgery	1877	48 (2.6)	7 (0.4)	95 (5.1)	1381
Arteriovenous malformation*	110	0	0	0	105
Pulmonary sequestration	124	0	0	0	108
Postoperative bleeding ⋅ air leakage	590	18 (3.1)	1 (0.2)	47 (8.0)	394
Chylothorax	54	0	0	0	43
Others	999	30 (3.0)	6 (0.6)	48 (4.8)	731

(), Mortality %

A total of 127 lung transplantations were performed in 2023 which increased by 17% compared to 109 in 2022 [5] (Table 27), among which 102 and 17 were from brain-dead and living-related donors, respectively. 30-day mortality for total lung transplantation was 3.1% (4/127).

**Table 27:** Lung transplantation

	Cases	30-Day mortality		Hospital mortality
		Hospital	After discharge	
Single lung transplantation from brain-dead donor	40	0	0	0
Bilateral lung transplantation from brain-dead donor	78	0	0	3 (3.8)
Lung transplantation from living donor	9	0	0	1 (11.1)
Total lung transplantation	127	0	0	4 (3.1)
Donor of living donor lung transplantation	17	0	0	0
Donor of brain-dead donor lung transplantation	102			

(), Mortality %

In 2023, the number of VATS procedures increased by 5.6% from 77,405 to 80,320 compared to that of 2022. The population of VATS procedures in all procedures 87% in 2023 was similar to that in 2022 (87%) (Table 28).

**Table 28:** Video-assisted thoracic surgery

	Cases	30-Day mortality		Hospital mortality
		Hospital	After discharge	
11. Video-assisted thoracic surgery	80,320	276 (0.3)	87 (0.1)	525 (0.7)

(), Mortality % (including thoracic sympathectomy 236)

A total of 587 tracheobronchoplasty procedures were performed in 2023, including 320 sleeve lobectomies, 9 carinal reconstructions, and 6 sleeve pneumonectomies (Table 29). Hospital mortality rates for sleeve lobectomy, carinal reconstruction, and sleeve pneumonectomy were 1.3, 11.1, and 16.7% respectively.

**Table 29:** Tracheobronchoplasty

	Cases	30-Day mortality		Hospital mortality
		Hospital	After discharge	
12. Tracheobronchoplasty	587	4 (0.7)	1 (0.2)	11 (1.9)
Trachea	34	0	0	0
Sleeve resection with reconstruction	22	0	0	0
Wedge with simple closure	1	0	0	0
Wedge with patch closure	0	0	0	0
Total laryngectomy with tracheostomy	0	0	0	0
Others	11	0	0	0
Carinal reconstruction	9	1 (11.1)	0	1 (11.1)
Sleeve pneumonectomy	6	1 (16.7)	0	1 (16.7)
Sleeve lobectomy	320	1 (0.3)	1 (0.3)	4 (1.3)
Sleeve segmental excision	23	0	0	0
Bronchoplasty without lung resection	22	0	0	1 (4.5)
Others	173	1 (0.6)	0	4 (2.3)

(), Mortality %

A total of 359 pediatric surgeries were performed in 2023 with hospital mortality of 3.3% (Table 30).

**Table 30:** Pediatric surgery

	Cases	30-Day mortality		Hospital mortality
		Hospital	After discharge	
13. Pediatric surgery	359	12 (3.3)	0	12 (3.3)

(), Mortality %

Overall, 1193 combined resections of the neighboring organ(s) had been performed for primary lung cancer and mediastinal tumor in 2023. The combined resection for primary lung cancer includes 261, 101, 56, 50, 14, 6, 6, and 4 cases of the chest wall, pulmonary artery, pericardium, diaphragm, left atrium, aorta, brachiocephalic vein, and superior vena cava resections, respectively. The combined resection for mediastinal tumors includes 476, 341, 109, 67, 43, and 10 cases of lung, pericardium, brachiocephalic vein, superior vena cava, diaphragm, and chest wall resections, respectively (Table 31).

**Table 31:** Combined resection of neighboring organ(s)

	Cases	30-Day mortality		Hospital mortality
		Hospital	After discharge	
14. Combined resection of neighboring organ(s)	1193	13 (1.1)	0	21 (1.8)

Organ resected	Cases	30-Day mortality		Hospital mortality
		Hospital	After discharge	
A Primary lung cancer				
Aorta	6	0	0	0
Superior vena cava	4	1 (25.0)	0	2 (50.0)
Brachiocephalic vein	6	0	0	0
Pericardium	56	2 (3.6)	0	3 (5.4)
Pulmonary artery	101	1 (1.0)	0	2 (2.0)
Left atrium	14	1 (7.1)	0	1 (7.1)
Diaphragm	50	1 (2.0)	0	3 (6.0)
Chest wall (including ribs)	261	5 (1.9)	0	9 (3.4)
Vertebra	9	0	0	0
Esophagus	1	0	0	0
Total	508	11 (2.2)	0	20 (3.9)
B. Mediastinal tumor				
Aorta	2	0	0	0
Superior vena cava	67	0	0	1 (1.5)
Brachiocephalic vein	109	0	0	1 (0.9)
Pericardium	341	1 (0.3)	0	3 (0.9)
Pulmonary artery	3	0	0	0
Left atrium	1	0	0	0
Diaphragm	43	0	0	0
Chest wall (including ribs)	10	0	0	0
Vertebra	3	0	0	0
Esophagus	4	0	0	0
Lung	476	2 (0.4)	0	2 (0.4)
Total	1059	3 (0.3)	0	7 (0.7)

(), Mortality %

A total of 611 operations of lung cancer invading the chest wall of the apex had been performed in 2023 with hospital mortality of 1.5% (Table 32).

**Table 32:** Operation of lung cancer invading the chest wall of the apex

	Cases	30-Day mortality		Hospital mortality
		Hospital	After discharge	
15. Operation of lung cancer invading the chest wall of the apex	611	3 (0.5)	1 (0.2)	9 (1.5)

(), Mortality %. Includes tumors invading the anterior apical chest wall and posterior apical chest wall (superior sulcus tumor, so called Pancoast type)

A total of 4,983 diagnostic procedures were performed in 2023 (Table 33).

**Table 33:** Diagnostic procedures

	Cases	30-Day mortality		Hospital mortality
		Hospital	After discharge	
Mediastinoscopic biopsy	219	0	1 (0.5)	1 (0.5)
Lung biopsy for diffuse parenchymal lung disease	522	5 (1.0)	0	5 (1.0)
Biopsy for lymph node, tumor and pleura	2813	22 (0.8)	22 (0.8)	43 (1.5)
Others	1429	52 (3.6)	5 (0.3)	106 (7.4)

(), Mortality %

### (C) Esophageal surgery

In 2018, the data collection method for esophageal surgery was modified from self-reports using questionnaire sheets following each institution belonging to the Japanese Association for Thoracic Surgery to an automatic package downloaded from the NCD in Japan. Consequently, the registry excluded data for non-surgical cases with esophageal diseases. Furthermore, data regarding the histological classification of malignant tumors, multiple primary cancers, and mortality rates for cases with combined resection of other organs could not be registered because they were not included in the NCD. Instead, detailed data regarding postoperative surgical and non-surgical complications were collected from the NCD. Moreover, data regarding surgeries for corrosive esophageal strictures and salvage surgeries for esophageal cancer had been exceptionally registered by participating institutions (Table 34).

**Table 34:** Benign esophageal diseases

		Operation( +)				T/L*3		
	Cases	Hospital mortality			Cases	Hospital mortality		
		~ 30 days	31–90 days	Total (including after 91 days mortality)		~ 30 days	31–90 days	Total (including after 91 days mortality)
1.Achalasia	161	0	0	0	46	0	0	0
2.Benign tumor	42	0	0	0	36	0	0	0
3.Diverticulum	32	0	0	0	12	0	0	0
4.Hiatal hernia	549	1 (0.2)	0	1 (0.2)	472	1 (0.2)	0	1 (0.2)
5.Spontaneous rupture of the esophagus	101	5 (5.0)	2 (2.0)	7 (6.9)	2	0	0	1 (50.0)
6.Esophago-tracheal fistula	5	1 (20.0)	0	1 (20.0)	0	0	0	0
7.Esophagitis, Esophageal ulcer	70	1 (1.4)	0	1 (1.4)	68	1 (1.5)	0	1 (1.5)
8.Corrosive stricture of the esophagus	7	0	0	0	3	0	0	0
Total	967	8 (0.8)	2 (0.2)	10 (1.0)	639	2 (0.3)	0	3 (0.5)

(), Mortality %

*T/L* Thoracoscopic and/or laparoscopic

**Fig. 3:**
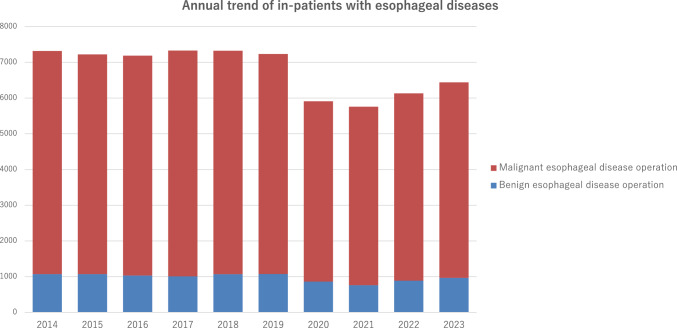
Annual trend of in-patients with esophageal diseases

Throughout 2023, 6439 patients underwent surgery for esophageal diseases (967 and 5472 for benign and malignant esophageal diseases, respectively) from institutions across Japan. Compared to 2019, there was a total decrease of 796 cases (11.0%) observed. These significant declines were largely influenced by the COVID-19 pandemic that began in 2020, with factors such as surgical restrictions, reduced medical visits, and postponed screenings being considered as contributing factors (Fig. 3). However, the number of esophageal surgeries in 2023 increased by 307 compared to 2022. As the issues related to COVID-19 are being resolved, a gradual recovery in the number of surgeries is expected in the future.

Concerning benign esophageal diseases (Table 34), thoracoscopic and/or laparoscopic surgeries were performed in 97.1% (68/70), 86% (472/549), 85.7% (36/42), and 28.6% (46/161) of patients with esophagitis (including esophageal ulcer), hiatal hernia, benign tumors, and achalasia, respectively. The decrease in the proportion of thoracoscopic and/or laparoscopic surgeries for achalasia is likely due to the gradual adoption of peroral endoscopic myotomy (POEM) in Japan. Conversely, 98.0% (99/101) of patients with spontaneous rupture of the esophagus underwent open surgery. Hospital mortality rates within 30 postoperative days were 0.2% (1/549), 5% (5/101) for hiatal hernia and spontaneous rupture of the esophagus, respectively.

The most common tumor location for malignant esophageal diseases was the thoracic esophagus (Table 35). Among the cases with esophageal malignancies, esophagectomy for superficial and advanced cancers was performed in 2010 (36.7%) and 3462 (63.3%), respectively. Hospital mortality rates within 30 days after esophagectomy were 0.3% and 0.6% for patients with superficial and advanced cancer, respectively.

**Table 35:** Malignant esophageal disease

	Operation( +)				Thoracoscopic and/or laparscopic procedure					
	Cases	Hospital mortality			Cases	Conversion to thoracotomy	Hospital mortality			
		~ 30 days	31–90 days	Total (including after 91 days mortality)			~ 30 days		31–90 days	Total (including after 91 days mortality)
Location										
(1) Cervical esophagus	128	1 (0.8)	2 (1.6)	3 (2.3)	69	0	1 (1.4)		1 (1.4)	2 (2.9)
(2) Thoracic esophagus	4449	23 (0.5)	15 (0.3)	38 (0.9)	4290	17 (0.4)	20 (0.5)		12 (0.3)	32 (0.7)
( 3) Abdominal esophagus	517	1 (0.2)	4 (0.8)	5 (1.0)	455	0	1 (0.2)		3 (0.7)	4 (0.9)
Total	5094	25 (0.5)	21 (0.4)	46 (0.9)	4814	17 (0.4)	22 (0.5)		16 (0.3)	38 (0.8)
Tumor depth										
(A)Superficial cancer(T1)										
(1) Transhiatal esophagectomy	4	0	0	0	0	0	0		0	0
(2) Mediastinoscopic esophagectomy and reconstruction	83	1 (1.2)	0	1 (1.2)	83	0	1 (1.2)		0	1 (1.2)
(3) Transthoracic (rt.) esophagectomy and reconstruction	1010	4 (0.4)	1 (0.1)	5 (0.5)	969	2 (0.2)	4 (0.4)		1 (0.1)	5 (0.5)
(4) Transthoracic (lt.) esophagectomy and reconstruction	10	0	0	0	6	0	0		0	0
(5) Cervical esophageal resection and reconstruction	20	0	0	0	0	0	0		0	0
(6) Robot-assisted esophagectomy and reconstruction	699	1 (0.1)	2 (0.3)	3 (0.4)	699	1 (0.1)	1 (0.1)		2 (0.3)	3 (0.4)
(7) Others	11	0	0	0	0	0	0		0	0
(8) Esophagectomy without reconstruction	173	0	1 (0.6)	1 (0.6)	47	24 (51.1)	0		1 (2.1)	1 (2.1)
Subtotal	2010	6 (0.3)	4 (0.2)	10 (0.5)	1804	27 (1.5)	6 (0.3)		4 (0.2)	10 (0.6)
(B)Advanced cancer(T2-T4)										
(1) Transhiatal esophagectomy	6	0	1 (16.7)	1 (16.7)	0	0	0		0	0
(2) Mediastinoscopic esophagectomy and reconstruction	127	1 (0.8)	1 (0.8)	2 (1.6)	127	0	1 (0.8)		1 (0.8)	2 (1.6)
(3) Transthoracic (rt.) esophagectomy and reconstruction	1870	12 (0.6)	11 (0.6)	22 (1.2)	1773	14 (0.8)	10	(0.6)	8 (0.5)	7 (0.4)
(4) Transthoracic (lt.) esophagectomy and reconstruction	38	2 (5.3)	1 (2.6)	3 (7.9)	19	0	1 (5.3)		0	1 (5.3)
(5) Cervical esophageal resection and reconstruction	45	0	0	0	0	0	0		0	0
(6) Robot-assisted esophagectomy and reconstruction	1135	4 (0.4)	4 (0.4)	8 (0.7)	1135	0	4 (0.4)		4 (0.4)	8 (0.7)
(7) Others	27	0	0	0	1	0	0		0	0
(8) Esophagectomy without reconstruction	214	3 (1.4)	0	4 (1.9)	73	27 (37.0)	3 (4.1)		2 (2.7)	14 (19.2)
Subtotal	3462	22 (0.6)	18 (0.5)	40 (1.2)	3128	41 (1.3)	19 (0.6)		15 (0.5)	32 (1.0)
Total	5472	28 (0.5)	22 (0.4)	50 (0.9)	4932	68 (1.4)	25 (0.5)		19 (0.4)	42 (0.9)

	Cases	Overall morbidity	Morbidity ≥ CD III		Surgical complications					
					Surgical site infection			Anastomotic leakage	Recurrent nerve palsy	Wound dehiscence
					Superficial incision	Deep incision	Organ space			
Location										
(1) Cervical esophagus	128	76 (59.4)	42 (32.8)		7 (5.5)	2 (1.6)	9 (7.0)	16 (12.5)	16 (12.5)	3 (2.3)
(2) Thoracic esophagus	4449	2499 (56.2)	950 (21.4)		283 (6.4)	114 (2.6)	296 (6.7)	485 (10.9)	579 (13.0)	41 (0.9)
(3) Abdominal esophagus	517	257 (49.7)	110 (21.3)		32 (6.2)	18 (3.5)	45 (8.7)	63 (12.2)	46 (8.9)	6 (1.2)
Total	5094	2832 (55.6)	1102 (21.6)		322 (6.3)	134 (2.6)	350 (6.9)	564 (11.1)	641 (12.6)	50 (1.0)
Tumor depth										
(A)Superficial cancer (T1)										
(1) Transhiatal esophagectomy	4	3 (75.0)	3 (75.0)		0	0	1 (25.0)	1 (25.0)	0	0
(2) Mediastinoscopic esophagectomy and reconstruction	83	48 (57.8)	20 (24.1)		6 (7.2)	2 (2.4)	5 (6.0)	9 (10.8)	22 (26.5)	2 (2.4)
(3) Transthoracic (rt.) esophagectomy and reconstruction	1010	546 (54.1)	197 (19.5)		68 (6.7)	31 (3.1)	76 (7.5)	120 (11.9)	102 (10.1)	10 (1.0)
(4) Transthoracic (lt.) esophagectomy and reconstruction	10	2 (20.0)	1 (10.0)		0	0	0	0	1 (10.0)	0
(5) Cervical esophageal resection and reconstruction	20	9 (45.0)	5 (25.0)		2 (10.0)	0	0	0	5 (25.0)	1 (5.0)
(6) Robot-assisted esophagectomy and reconstruction	699	380 (54.4)	167 (23.9)		48 (6.9)	22 (3.1)	50 (7.2)	86 (12.3)	86 (12.3)	8 (1.1)
(7) Others	11	3 (27.3)	1 (9.1)		0	0	0	0	0	0
(8) Esophagectomy without reconstruction	173									
Subtotal	2010	991 (49.3)	394 (19.6)		124 (6.2)	55 (2.7)	132 (6.6)	216 (10.7)	216 (10.7)	21 (1.0)
(B)Advanced cancer (T2–T4)										
(1) Transhiatal esophagectomy	6	5 (83.3)	1 (16.7)		0	0	0	1 (16.7)	0	0
(2) Mediastinoscopic esophagectomy and reconstruction	127	82 (64.6)	30 (23.6)		9 (7.1)	5 (3.9)	9 (7.1)	15 (11.8)	32 (25.2)	3 (2.4)
(3) Transthoracic (rt.) esophagectomy and reconstruction	1870	1064 (56.9)	393 (21.0)		118 (6.3)	43 (2.3)	128 (6.8)	203 (10.9)	233 (12.5)	13 (0.7)
(4) Transthoracic (lt.) esophagectomy and reconstruction	38	17 (44.7)	6 (15.8)		0	0	4 (10.5)	5 (13.2)	3 (7.9)	1 (2.6)
(5) Cervical esophageal resection and reconstruction	45	33 (73.3)	21 (46.7)		6 (13.3)	3 (6.7)	4 (8.9)	5 (11.1)	8 (17.8)	2 (4.4)
(6) Robot-assisted esophagectomy and reconstruction	1135	622 (54.8)	251 (22.1)		65 (5.7)	28 (2.5)	67 (5.9)	113 (10.0)	151 (13.3)	10 (0.9)
(7) Others	27	15 (55.6)	6 (22.2)		0	0	6 (22.2)	6 (22.2)	0	0
(8) Esophagectomy without reconstruction	214									
Subtotal	3462	1838 (53.1)	708 (20.5)		198 (5.7)	79 (2.3)	218 (6.3)	348 (10.1)	427 (12.3)	29 (0.8)
Total	5472	2829 (51.7)	1102 (20.1)		322 (5.9)	134 (2.4)	350 (6.4)	564 (10.3)	643 (11.8)	50 (0.9)

	Cases	Nonsurgical complications									Readmission within 30d	Reoperation within 30d
		Pneumonia	Unplanned intubation	Prolonged ventilation > 48 h	Pulmonary embolism	Atelectasis	Renal failure	CNS events	Cardiac events	Septic shock		
Location												
(1) Cervical esophagus	128	16 (12.5)	8 (6.3)	11 (8.6)	0	6 (4.7)	2 (1.6)	0	1 (0.8)	2 (1.6)	0	16 (12.5)
(2) Thoracic esophagus	4449	700 (15.7)	159 (3.6)	164 (3.7)	41 (0.9)	172 (3.9)	12 (0.3)	17 (0.4)	10 (0.2)	35 (0.8)	129 (2.9)	227 (5.1)
(3) Abdominal esophagus	517	66 (12.8)	13 (2.5)	16 (3.1)	8 (1.5)	23 (4.4)	2 (0.4)	2 (0.4)	1 (0.2)	2 (0.4)	13 (2.5)	30 (5.8)
Total	5094	782 (15.4)	180 (3.5)	191 (3.7)	49 (1.0)	201 (3.9)	16 (0.3)	19 (0.4)	12 (0.2)	39 (0.8)	142 (2.8)	273 (5.4)
Tumor depth												
(A)Superficial cancer (T1)												
(1) Transhiatal esophagectomy	4	0	1 (25.0)	0	0	0	1 (25.0)	0	0	0	0	0
(2) Mediastinoscopic esophagectomy and reconstruction	83	12 (14.5)	2 (2.4)	3 (3.6)	1 (1.2)	2 (2.4)	0	0	0 (0.0)	0	1 (1.2)	4 (4.8)
(3) Transthoracic (rt.) esophagectomy and reconstruction	1010	148 (14.7)	41 (4.1)	44 (4.4)	14 (1.4)	37 (3.7)	5 (0.5)	2 (0.2)	2 (0.2)	6 (0.6)	28 (2.8)	47 (4.7)
(4) Transthoracic (lt.) esophagectomy and reconstruction	10	0	0	0	0	0	0	0	0	0	0	0
(5) Cervical esophageal resection and reconstruction	20	1 (5.0)	2 (10.0)	1 (5.0)	0	0	0	0	0	0	0	2 (10.0)
(6) Robot-assisted esophagectomy and reconstruction	699	86 (12.3)	11 (1.6)	11 (1.6)	9 (1.3)	28 (4.0)	1 (0.1)	5 (0.7)	0	3 (0.4)	21 (3.0)	38 (5.4)
(7) Others	11	0	0	0	0	0	0	0	0	0	0	1 (9.1)
(8) Esophagectomy without reconstruction	173											
Subtotal	2010	247 (12.3)	57 (2.8)	59 (2.9)	24 (1.2)	67 (3.3)	7 (0.3)	7 (0.3)	2 (0.1)	9 (0.4)	50 (2.5)	92 (4.6)
(B)Advanced cancer (T2-T4)												
(1) Transhiatal esophagectomy	6	1 (16.7)	0	1 (16.7)	0	0	0	0	0	0	0	1 (16.7)
(2) Mediastinoscopic esophagectomy and reconstruction	127	12 (9.4)	3 (2.4)	2 (1.6)	2 (1.6)	2 (1.6)	0	3 (2.4)	0	1 (0.8)	3 (2.4)	7 (5.5)
(3) Transthoracic (rt.) esophagectomy and reconstruction	1870	320 (17.1)	77 (4.1)	86 (4.6)	15 (0.8)	80 (4.3)	4 (0.2)	6 (0.3)	7 (0.4)	17 (0.9)	49 (2.6)	103 (5.5)
(4) Transthoracic (lt.) esophagectomy and reconstruction	38	5 (13.2)	4 (10.5)	4 (10.5)	1 (2.6)	1 (2.6)	1 (2.6)	1 (2.6)	1 (2.6)	3 (7.9)	0	4 (10.5)
(5) Cervical esophageal resection and reconstruction	45	6 (13.3)	2 (4.4)	3 (6.7)	0	5 (11.1)	1 (2.2)	1 (2.2)	1 (2.2)	1 (2.2)	0	10 (22.2)
(6) Robot-assisted esophagectomy and reconstruction	1135	188 (16.6)	36 (3.2)	34 (3.0)	7 (0.6)	44 (3.9)	3 (0.3)	1 (0.1)	1 (0.1)	8 (0.7)	39 (3.4)	56 (4.9)
(7) Others	27	3 (11.1)	1 (3.7)	1 (3.7)	0	2 (7.4)	0	0	0	0	1 (3.7)	0
(8) Esophagectomy without reconstruction	214											
Subtotal	3462	535 (15.5)	123 (3.6)	131 (3.8)	25 (0.7)	134 (3.9)	9 (0.3)	12 (0.3)	10 (0.3)	30 (0.9)	92 (2.7)	181 (5.2)
Total	5472	782 (14.3)	180 (3.3)	190 (3.5)	49 (0.9)	201 (3.7)	16 (0.3)	19 (0.3)	12 (0.2)	39 (0.7)	142 (2.6)	273 (5.0)

**Fig. 4:**
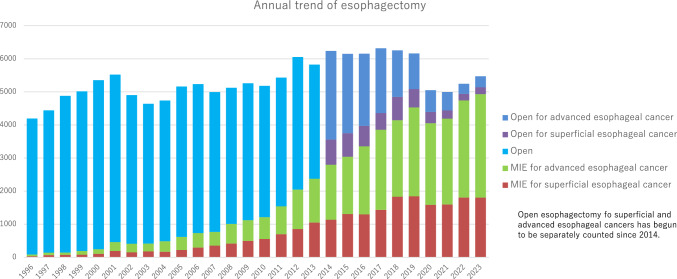
Annual trend of esophagectomy

Among esophagectomy procedures, transthoracic esophagectomy via right thoracotomy or right thoracoscopy was most commonly adopted for patients with superficial (1010/2010, 50.2%) and advanced cancer (1870/3462, 54.0%) (Table 35). Transhiatal esophagectomy, which is commonly performed in Western countries, was adopted in only 4 (0.2%) and 6 (0.2%) patients with superficial and advanced cancer who underwent esophagectomy in Japan, respectively. Minimally invasive esophagectomy (MIE) including thoracoscopic and/or laparoscopic esophagectomy, robot-assisted esophagectomy and mediastinoscopic esophagectomy was utilized in 1804 (89.8%) and 3128 (90.4%) patients with superficial and advanced cancer, respectively. Incidence of MIE for superficial or advanced cancer has been increasing, whereas that of open surgery, especially for advanced cancer, has been decreasing annually (Fig. 4). Although mediastinoscopic esophagectomy was performed only for 83 (4.1%) and 127 (3.7%) patients with superficial and advanced esophageal cancer, respectively. Robot-assisted esophagectomy has remarkably increased since 2018 when insurance approval was obtained in Japan, and performed for 699 (34.8%) and 1135 (32.8%) patients with superficial and advanced esophageal cancer, respectively in 2023. Patients who underwent robot-assisted surgery are increasing for both superficial and advancer esophageal cancers (33.1% and 37.6% increases compared to that in 2022, respectively). Hospital mortality rates within 30 days after MIE were 0.3% and 0.6% for patients with superficial and advanced cancer, respectively (Table 35).

Detailed data collection regarding postoperative surgical and non-surgical complications was initiated in 2018. Overall, 1102 (20.1%) of 5472 patients developed grade III or higher complications based on the Clavien–Dindo classification in 2023 (Table 35). The incidence of grade III or higher complications was relatively higher in cervical esophageal cancer compared to thoracic or abdominal esophageal cancer. Among surgical complications in patients with advanced esophageal cancer, anastomotic leakage, and recurrent nerve palsy occurred in 10.9% and 12.5% of the patients who underwent right transthoracic esophagectomy, in 10% and 13.3% of those who underwent robot-assisted esophagectomy, and in 11.8% and 25.2% of those who underwent mediastinoscopic esophagectomy, respectively. Among non-surgical postoperative complications, pneumonia occurred in 14.3% of the patients, 3.3% of whom underwent unplanned intubation. Postoperative pulmonary embolism occurred in 0.9% of the patients. These complication rates, including the others, were similar to those in 2022.

We aim to continue our efforts in collecting comprehensive survey data through more active collaboration with the Japan Esophageal Society and other related institutions.

## Data Availability

Based on the data use policy of JATS, data access is approved through assessment by the JATS: Committee for Scientific Affairs. Those interested in using the data should contact the JATS: Committee for Scientific Affairs(survey@jpats.org) to submit a proposal. The use of the data is granted for the approved study proposals.
